# An unexpected Scalopini mole (Talpidae, Mammalia) from the Pliocene of Europe sheds light on the phylogeny of talpids

**DOI:** 10.1038/s41598-025-10396-1

**Published:** 2025-07-10

**Authors:** Adriana Linares-Martín, Marc Furió, Bruno Gómez de Soler, Jordi Agustí, Oriol Oms, Federica Grandi, Hugues-Alexandre Blain, Elena Moreno-Ribas, Pedro Piñero, Gerard Campeny

**Affiliations:** 1 Institut Català de Paleoecologia Humana i Evolució Social (IPHES-CERCA), Zona Educacional 4, Campus Sescelades URV (Edifici W3), Tarragona, 43007 Spain; 2 Departament d’Història i Història de l’Art, Universitat Rovira i Virgili (URV), Avinguda de Catalunya 35, Tarragona, 43002 Spain; 3 Departament de Geologia, Universitat Autònoma de Barcelona (UAB), Cerdanyola del Vallès, Barcelona, 08193 Spain; 4 Present Address: Institut Català de Paleontologia Miquel Crusafont (ICP-CERCA), Edifici Z, c/ de les Columnes, Campus de la UAB, Cerdanyola del Vallès, Barcelona, 08193 Spain; 5 Departament de Botànica i Geologia, Universitat de València, Doctor Moliner 50, Burjassot, 46100 Spain

**Keywords:** Camp dels Ninots, Maar, Konservat-Lagerstätten, Spain, Fossorial, Palaeontology, Phylogenetics, Taxonomy

## Abstract

The Pliocene Konservat-Lagerstätten maar lake site of Camp dels Ninots (NE Iberian Peninsula) has recently delivered a partial skeleton of a mole (family Talpidae) with many elements in anatomical connection. At a first glance, molar and humerus size, geological time interval, and geographical location suggested that this specimen could correspond to *Talpa minor*. However, after some mechanical preparation of the clay block (matrix removal, consolidation, and cleaning) and a micro-CT scan, this excellently preserved specimen turned out to be an unknown species to science. The resulting 3D models of this new form, *Vulcanoscaptor ninoti* gen. et sp. nov., revealed some peculiar morphological traits in teeth, mandible, and postcranial elements, which according to the phylogenetic analysis carried out, would allocate this new species within the tribe Scalopini. This is surprising, because the closest relatives of the new species within this tribe live nowadays in North America, and only some related taxa had been previously reported in the Oligocene and Miocene fossil record from Europe. The postcranial construction of this specimen reveals a highly fossorial lifestyle supported by a complex forelimb structure. How such a specialized digging animal reached the maar lake sediments where it was finally preserved is still to be solved. Some hypotheses consider swimming abilities for this extinct species. Alternatively, this specimen could be the remaining portions of a floated or scavenged carcass whose remains fell into the lake and reached the anoxic bottom.

**Supplementary Information:**

The online version contains supplementary material available at 10.1038/s41598-025-10396-1.

## Introduction

The Talpidae are probably one of the most intriguing families of mammals from the paleobiogeographic point of view. Only a few species within this group can be considered truly cursorial, because most of them show in some extent adaptations to fossorial activity. Indeed, many talpids are extremely adapted to subterranean and aquatic lifestyles, and their subaerial movements or out of water displacements are rather clumsy. Out of tunnels or rivers, most species of moles are highly vulnerable to potential predators. Thus, anyone would expect these animals to follow a rather predictable and constant (paleo-)biogeographic evolutionary history, with no significant migrations, considering all the possible physical and biological barriers that could constrain their distribution. Surprisingly, the fossil record is rather indicating a quite different story.

The genus *Eotalpa* is the oldest fossorial representative of the family known hitherto^1,2^. According to the fossil record of the younger representatives, few groups have originated and stayed in the same region that they currently inhabit. For instance, Schwermann et al.^3^. conducted a thorough study on the evolutionary history of the Scalopini, concluding that numerous transcontinental colonization events occurred between Asia, Europe, and North America, to the extent that no unambiguous location for the origin of the group could be identified. The recent discovery of *Alpiscaptulus medogensis*, a living species of Scalopini from the Himalayas^4^has just added some more questions to this enigma.

In a similar way, uropsilines are nowadays represented only by the genus *Uropsilus* (but see disagreements in He et al.^5^) and restricted to Central and Southeastern Asia. The fossil record shows, however, that its Oligocene, Miocene and Pliocene relatives (*Desmanella*, *Mystipterus*, *Asthenoscapter*, *Mygatalpa*, *Theratiskos*) lived in European, Minor Asian and North American lands^6–11^.

Another example is found in the tribe Condylurini, nowadays being solely represented by the endemic North American star-nosed mole, *Condylura cristata*. However, unequivocal representatives of the genus have been found in the Pliocene of Poland^12,13^ and the Middle Miocene of Kazakhstan^14^. Likewise, *Neurotrichus gibbsii* is the only living representative of the tribe Neurotrichini, according to Hutterer^15^. The hypothesis of a North American origin of *Neurotrichus* judging by its solely current occurrence in this continent would apparently be parsimonious. Nonetheless, the cladistic analysis performed by Schwermann and Thompson^16^ suggested that this genus could find its closest living relative in the genus *Scaptonyx*, which mostly currently inhabits elevated areas from China. The extinct genus *Rzebikia* is a very similar form to *Neurotrichus*, but it is only found in Plio-Pleistocene sites from Europe^17^. Both genera, *Neurotrichus* and *Rzebikia* are putative descendants of an Asian form, *Quyania*^18^. Far from being simple, the most likely explanation to such distribution according to Sansalone et al.^17^ is that the original Asian stock of moles finally derived into *N. gibbsii* after colonizing North America in the Early Pliocene, while another one or two migration waves towards Eastern Europe could have resulted in several European forms.

The case of the swimming forms of the Desmanini is similarly puzzling. The Russian desman (*Desmana moschata*), an extant endemic species living in Russia, Ukraine and Kazakhstan, is the only survivor of a genus that apparently originated in the south of the Iberian Peninsula^19^. Ironically, the Iberian desman (*Galemys pyrenaicus*), an endemic form that currently inhabits some stream headwaters in this part of the European continent, belongs to a genus of unknown geographic origin. During the Late Neogene, the tribe had a much wider distribution, although restricted to Europe and parts of Asia Minor^20^.

Some of these apparent inconsistencies are probably rooted in a very limited view of the talpid fossil record. On the one hand, moles are rather conservative in their morphology, likely due to the constant conditions where they thrive. It could be that once acquired an efficient capacity for digging (or swimming), there were few possible morphological modifications to adapt to different lifestyles^21^. On the other hand, the fossil finds of talpids usually correspond to disarticulated and/or incomplete elements, not always easy to identify to the species level. In many cases, it is difficult to discriminate whether the (little) variation in size and morphology observed in the fossil assemblages is due to interspecific or intraspecific variability. A good example is found on the uncertainty of how many species of *Talpa* occur in the European fossil record, a debate that has been extended for decades in specialized literature^9,22,23^. For a comprehensive list of approaches to link humeri and dentitions in fossil talpids, the reader is referred to Van den Hoek Ostende and Fejfar^24^.

Fortunately, the fossil record is not always limited to patchy finds and fragmentary elements. In the last years, some fossil-Lagerstätten^25^ sites have provided several exceptional specimens with many skeletal elements that can be clearly ascribed to the same individual^16,26^. Such finds are two-fold precious. On the one hand, they allow checking useful morphological characters located in fragile parts, which are not frequently preserved in the fossil record. On the other hand, they link dental to postcranial elements, thus becoming very useful in the calculation of the relative sizes between both. Each one of these exceptional finds constitutes a reference milestone to which compare many other isolated fossil elements of talpids, mostly teeth and humeri.

In the present work, we study a new partial fossil skeleton of a burrowing mole that has been recently discovered in the Pliocene locality of Camp dels Ninots (CN) in NE Spain. This exceptional fossil find preserves several postcranial elements associated to almost complete dentitions and the whole mandible, all of them clearly belonging to the same individual. This specimen was tentatively ascribed to *Talpa minor* at a first glance, considering the chronology and location of the fossil site in combination to its general size. Nevertheless, our colleague Dr. Lars van den Hoek Ostende (Naturalis Biodiversity Center, Leiden, The Netherlands) noticed in a previous stage of the present work some morphological details against its ascription to the genus *Talpa*. The rest of the talpid genera hitherto identified in Pliocene sites from Spain^27^namely *Archaeodesmana*, *Desmana*, *Galemys*, and *Desmanella* could be easily discarded as well. Therefore, in the present study we aim to solve: (1) which is the species represented in this fossil site, (2) the phylogenetic position of this fossil among extant and extinct talpids, (3) how this species was adapted to excavate and live underground, and more specifically, (4) how this specimen reached the sedimentary environment where it was finally preserved.

## Geological settings

**Fig. 1:**
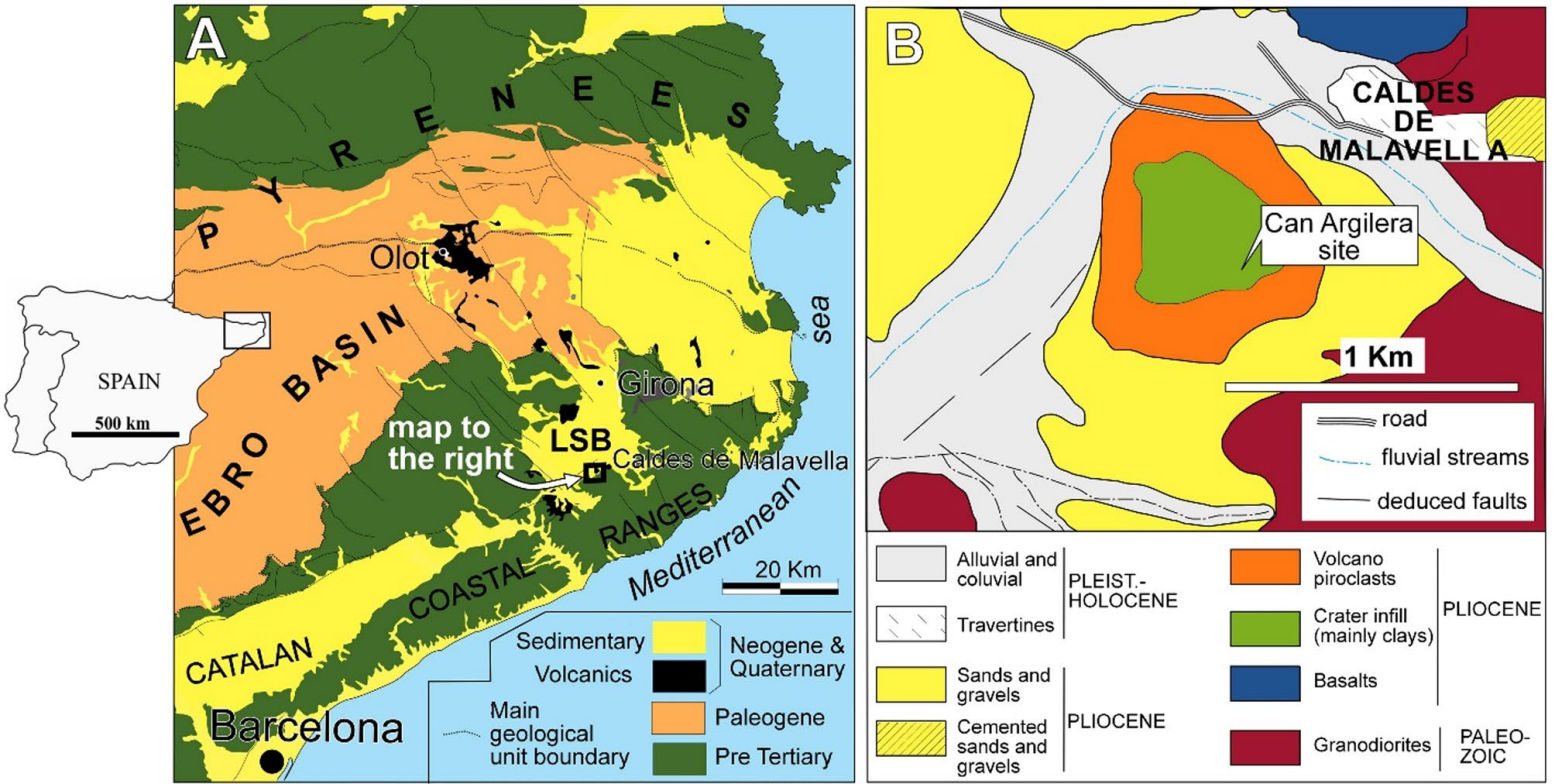
Geographical and geological context of the locality of CN and Can Argilera site. LSB: La Selva Basin. (Adapted from Gómez De Soler et al. 2012). Map generated with FreeHand™ 9.0 (https://macromedia-freehand.uptodown.com/windows)

CN is a Pliocene site located in the town of Caldes de Malavella, in the province of Girona, in the NE of the Iberian Peninsula (Fig. 1). Some analyses using magneto and cyclostratigraphic techniques had provided an age of ca. 3.25 Ma^28^. However, recent 40Ar/39Ar datation techniques are indicative of a much older age for the site, likely Early Pliocene (work in progress). The first fragmentary bone from this locality was found in 1985 and it was assigned to the genus *Leptobos* by Vicente^29^. Some later prospections resulted in new fossil inputs^30–34^ and in 2003 the Institut Català de Paleoecologia Humana i Evolució Social (IPHES-CERCA) started the systematic research projects and excavations. Since then, this locality has delivered several articulated skeletons of vertebrates of bovids (*Alephis tigneresi)*, tapirs (*Tapirus arvernensis)*, a rhinoceros (*Stephanorhinus* cf. *jeanvireti)*, snakes (*Natrix maura*), turtles (*Mauremys leprosa* and *Chelydropsis* cf. *pontica*), amphibians (Pleurodelinae indet., *Lissotriton* aff. *helveticus*, and *Pelophylax* sp.), and freshwater fishes (*Leuciscus* sp. and *Luciobarbus* sp.)^31–34^. To a lesser extent, also diatoms, invertebrates (insects), micro- and macroflora (such as *Laurophyllum sp.*) have been found^28,35,36^.

CN site was in origin a maar lake of a volcano found in the Catalan Volcanic Complex (CVC) at the southern border of La Selva basin. There, volcanic activity ranged from the Miocene till the Holocene. The CVC is part of the volcanic provinces of the European Rift System into the Catalan Coastal Ranges^37^ (Fig. 1). La Selva basin was the result of an extensional tectonic episode during the Miocene-Pliocene, generated by the movement of two sets of faults oriented ENE-WSW and NW-SE of a previously fractured Paleozoic basement (for further details about the geotectonic setting see Roca et al.^38^ and Tassone et al.^39^). The tectonic activity favored the volcanism during the Miocene, with intense phases during the Pliocene^40,41^. Several volcanic episodes are related with the meteoric water infiltration according to fractures of the basement and porosity of Pliocene materials, which filled the basin. The near surface magma-water interactions generated a mixed volcanism with an alternating eruptive-effusive phase. Phreatomagmatic vulcanism of explosive phases resulted in the formation of a maar crater^30,42^. Post volcanic sediments were accumulated within the maar crater as lacustrine deposits.

**Fig. 2:**
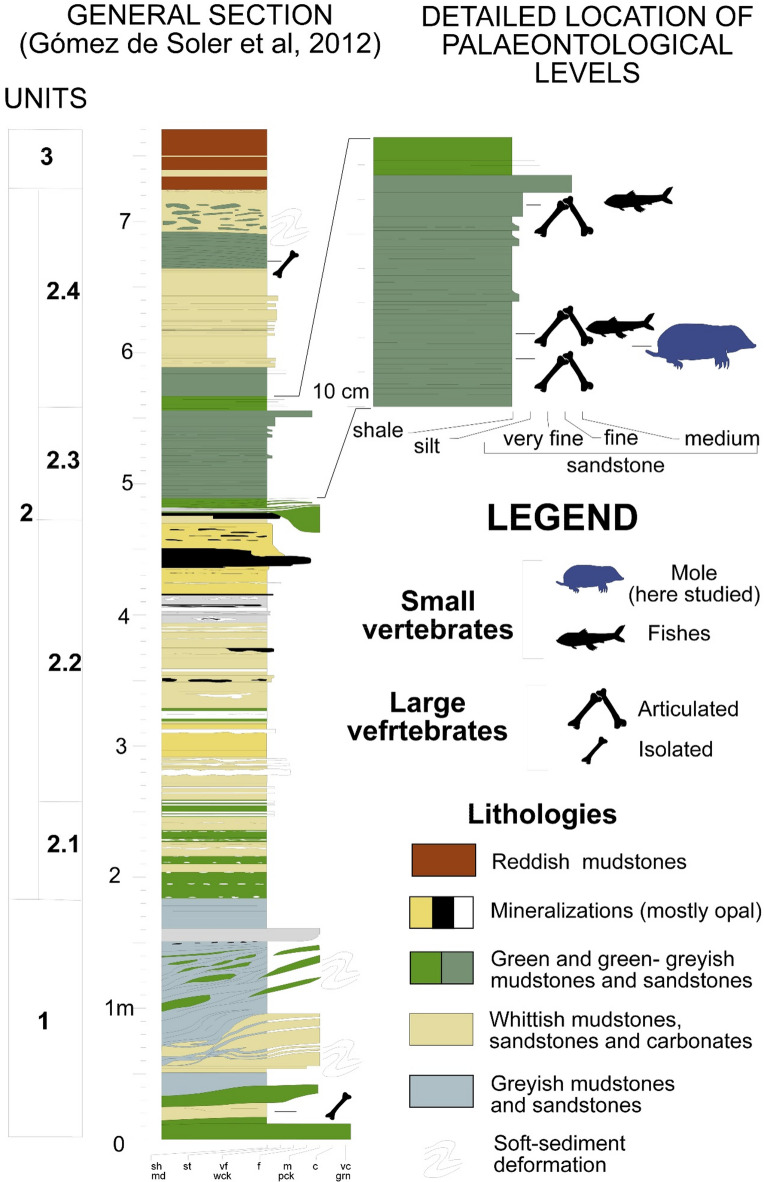
Stratigraphic section of Can Argilera. (Adapted from Gómez de Soler et al. 2012).

**Fig. 3:**
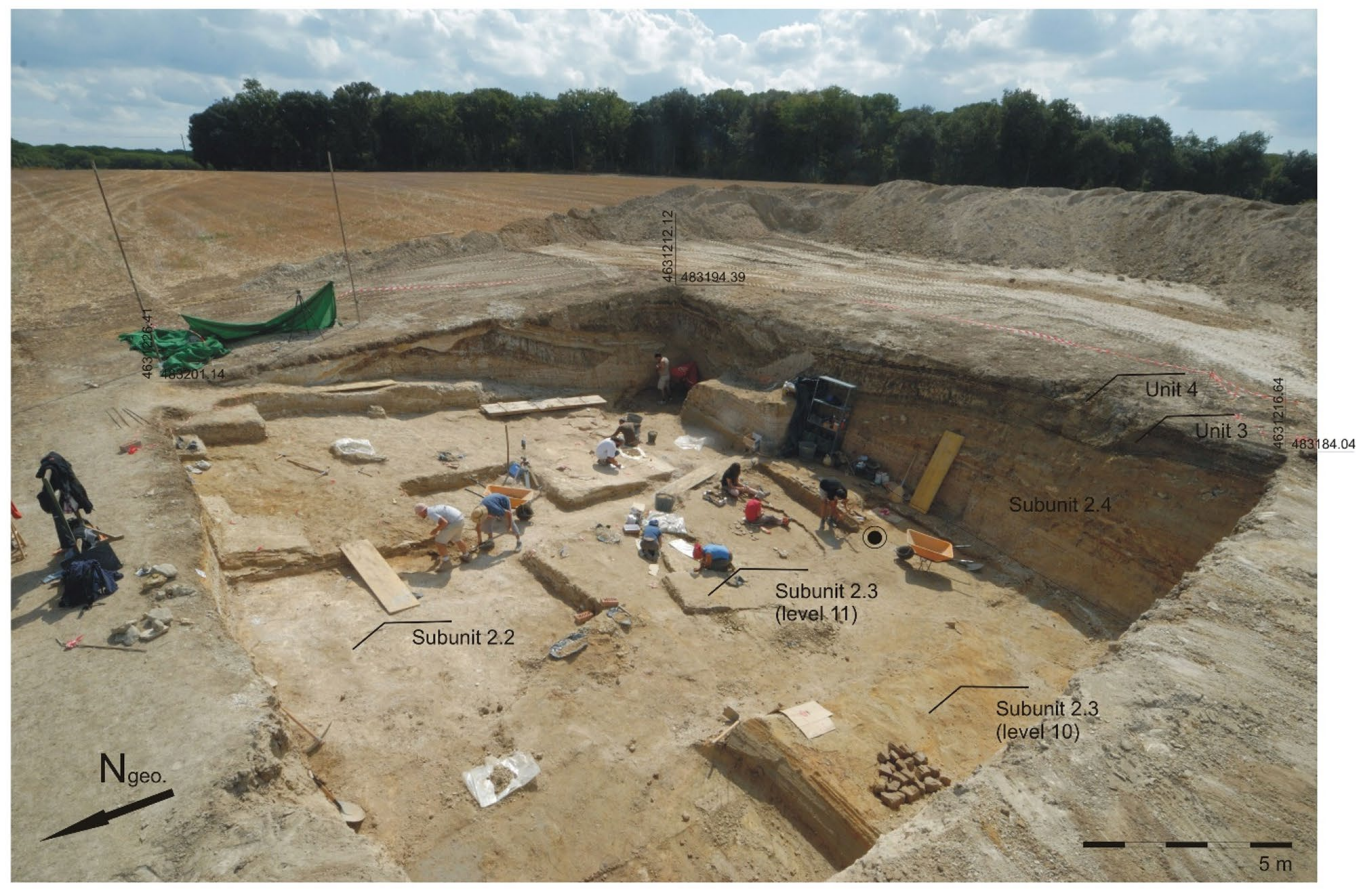
Excavation area of Can Argilera in CN (Pit 7/8). The exact place where the fossil specimen was found is indicated with a black spot.

The sedimentary infill of CN is formed by the typical vertical stratigraphic succession in maars^43,44^. The one described below is taken as a reference for this fossil site and represents a thick section of 8 meters at the Can Argilera excavation sector, with four units from its base to the top (Figs. 2 and 3). Unit 1 encompasses greyish clays, sandstones and diatomites. Unit 2 is represented by greenish laminated clays with diatoms, and it is differentiated in other four subunits; 2.1, 2.2 and 2.4 are characterized by the presence of carbonates. The subunit 2.3 (see ‘Detailed Location’ in Fig. 2) has been recorded in the Can Argilera sector and it is divided in fine-grained reddish sands with silty admixture (Layer 10) and lacustrine greenish silts with clay laminations (Layer 11). The latter subunit is the most remarkable one in terms of articulated skeletons of mammals and plant remains. Lastly, unit 3 is formed by reddish laminated clays and silty slope wash deposits. For further details on the specific geology of the site the reader is referred to Gómez de Soler et al.^31^Jiménez-Moreno et al.^28^Oms et al.^42^Rodríguez-Salgado et al.^45^.

## Results

### Systematic paleontology

Order Eulipotyphla Waddell, Okada & Hasegawa, 1999.

Family Talpidae Fischer, 1814.

Tribe Scalopini Gill, 1875.

Genus *Vulcanoscaptor* nov.

**Fig. 4:**
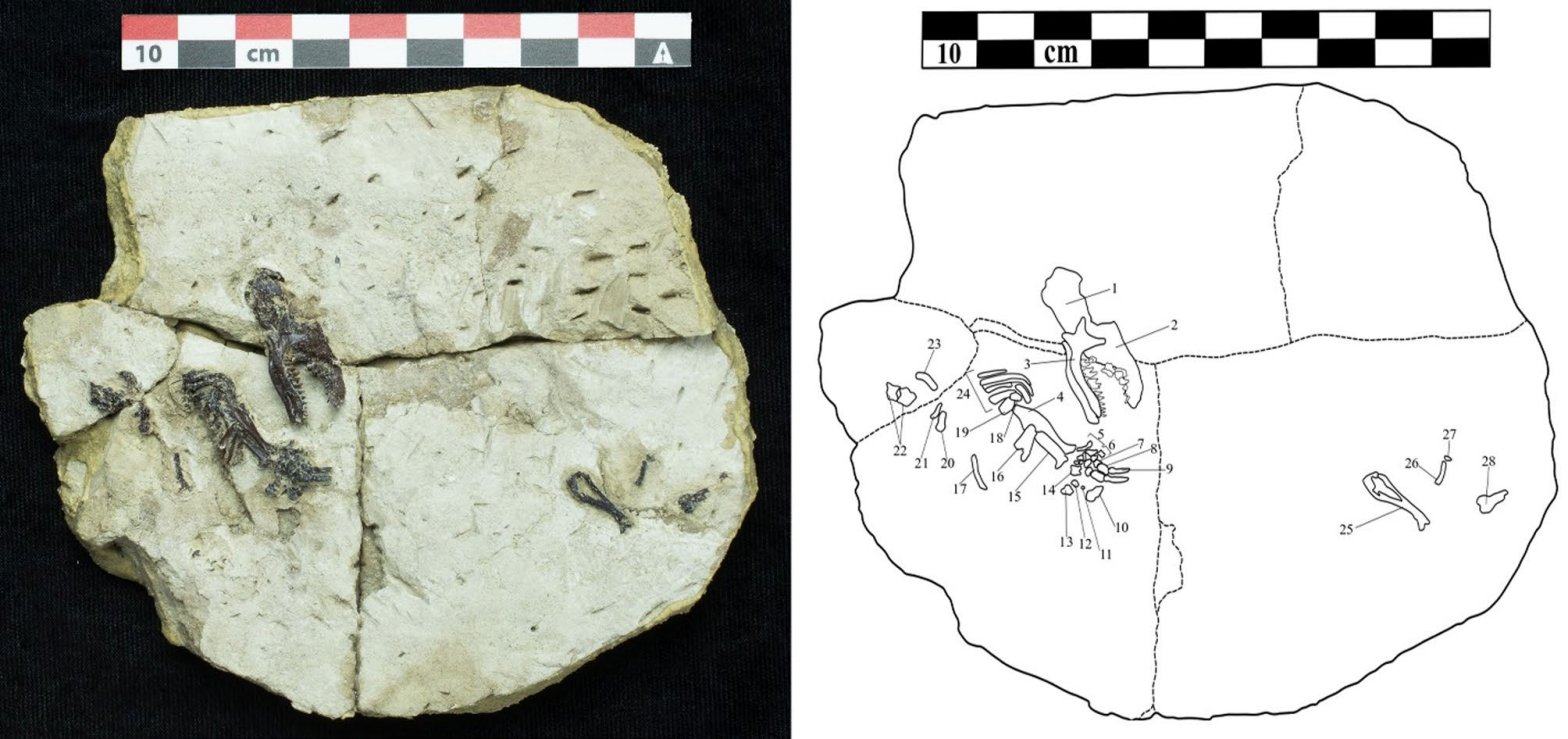
Partial skeleton of *Vulcanoscaptor ninoti* gen. et sp. nov. (CN10-O17-NIV11-12) from CN. Cranium (1), skull (2), mandibles (3), ulna (4), carpals (5,12,13), metacarpals (6,14), proximal phalanges (7), medial phalanges (8), distal phalanges (9,20), sesamoid bone (11,21), accessory sesamoid bone (10), radius (15), humerus (16), ribs (17,23,24), clavicle (18), scapula (19), vertebrae (22), tibiofibula (25), metatarsal (26), foot distal phalange (27), femur (28). Scale bar 10 cm.

**Fig. 5:**
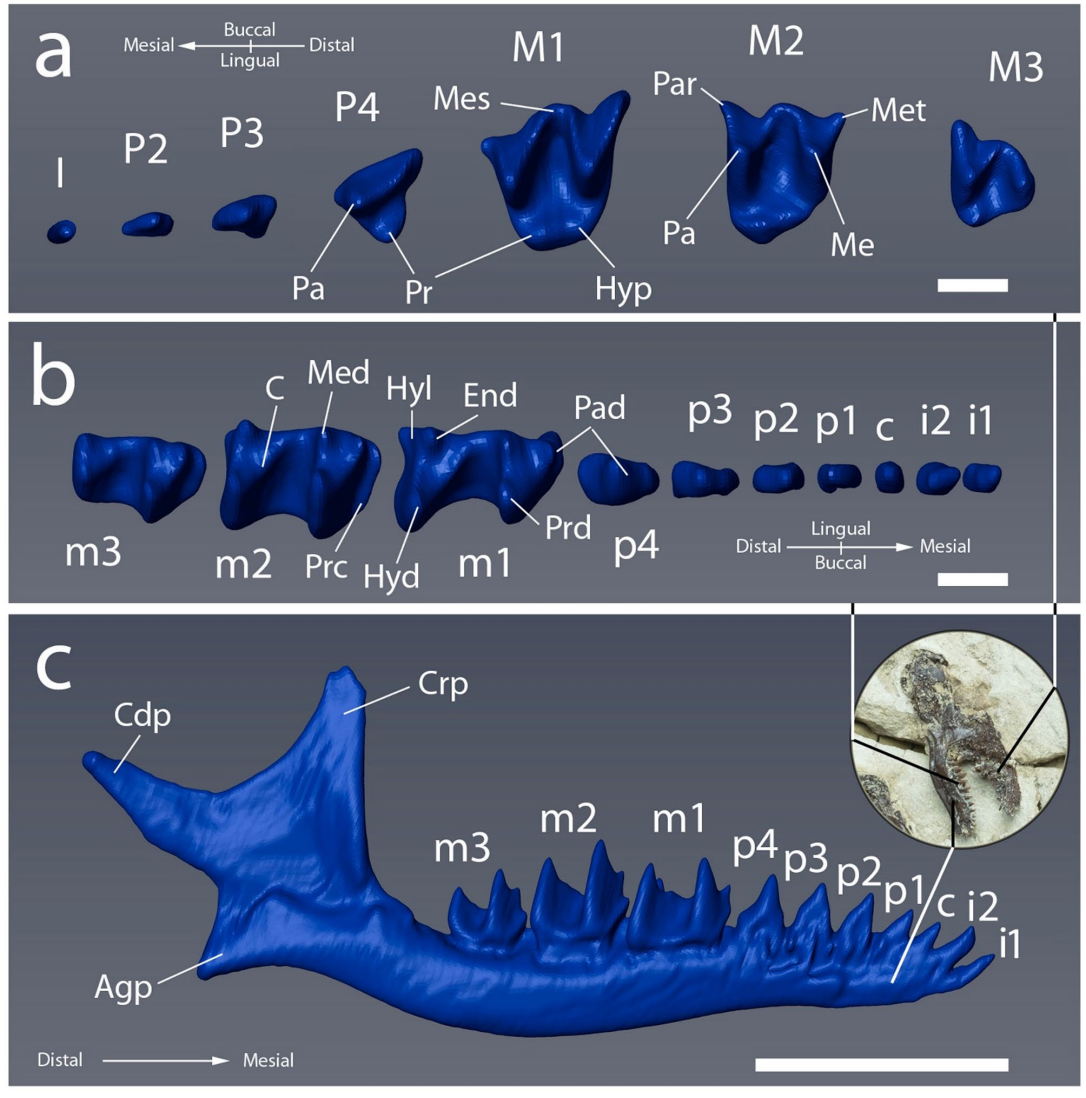
Digital 3D reconstruction of the dentognathic elements of *Vulcanoscaptor ninoti* gen. et sp. nov. (CN10-O17-NIV11-12). **a.** Left upper tooth-row (I3 + P2-M3) in occlusal view. **b.** Right lower tooth-row (m3-i2) in occlusal view. **c**. Right hemimandible in lateral view. Scale bar equals 1 mm in **a** and **b**, and 5 mm in **c**.

**Fig. 6:**
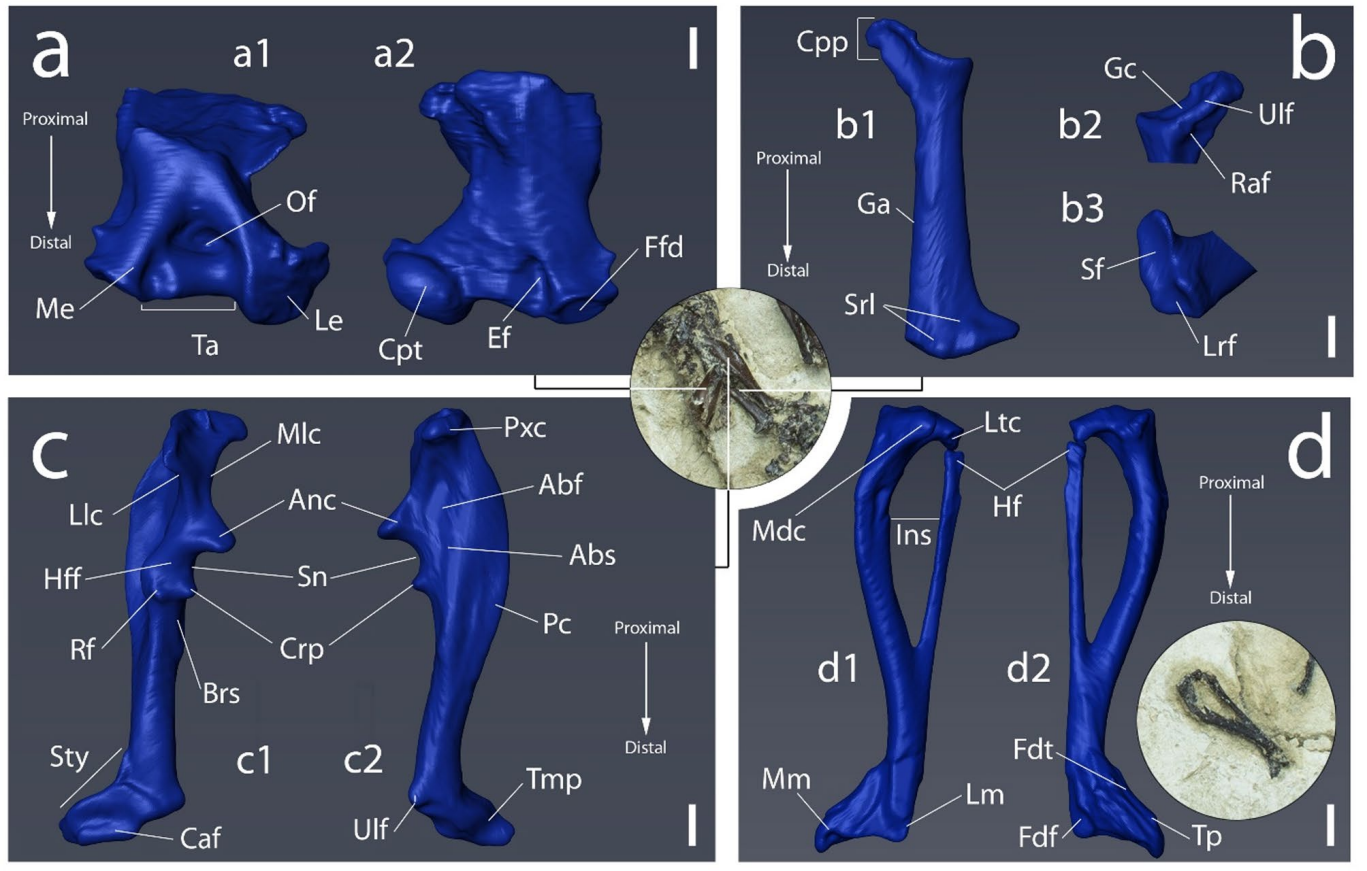
Digital 3D reconstruction of some forelimb elements of *Vulcanoscaptor ninoti* gen. et sp. nov. (CN10-O17-NIV11-12). **(a)** Right humerus; a1 posterior view; a2 anterior view. **(b)** Right radius: b1 lateral view; b2 proximal-medial view; b3 distal view. **(c)** Right ulna; c1 left anterior view; c2 lateral view. **(d)** Right tibiofibula; d1 anterior view; d2 posterior view. Scale bars at the corners equal 1 mm in all cases.

**Fig. 7:**
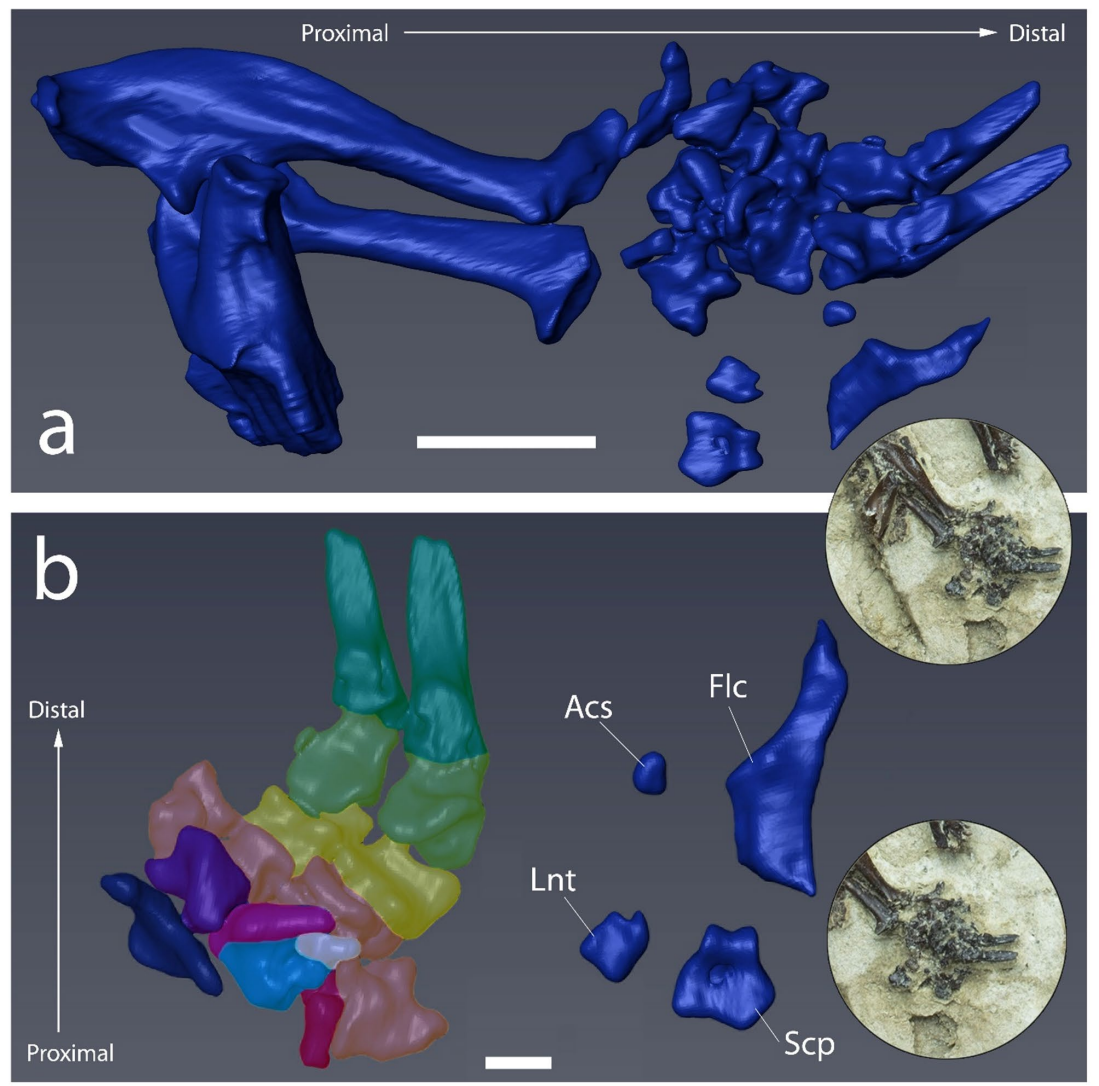
(**a**) Digital reconstruction of the right forelimb of *Vulcanoscaptor ninoti* gen. et sp. nov. (CN10-O17-NIV11-12) in its original position. (**b**) Detail of the right manus articulation in volar view. Carpals, metacarpals and phalanges are highlighted using different colors: triquetrum (dark blue), hamate (purple), capitate (pink), centrale (light blue), trapezoid (white), trapezium (red), metacarpals (orange), proximal phalanges (yellow), medial phalanges (light green), distal phalanges (dark green). Falciform, accessory sesamoid, lunate and scaphoid are disconnected from the hand. Scale bar equals 5 mm in (**a**) and1 mm in (**b**).

*Vulcanoscaptor ninoti* sp. nov. (Figures 4, 5, 6 and 7).

***Authorship of genus and species.*** Linares-Martín, 2025.

***Etymology.*** Name of the genus derived from the Latin word of ‘Vulcan’, the Roman god of fire, in reference to the volcanic nature of the source area, and ‘-scaptor’, from the ancient Greek word ‘scaptein’, to dig. Name of the species invoking ‘ninot’, the local word to refer the opaline nodules ‘doll-shaped’ typically found in the type-locality of the species, Camp dels Ninots.

***Holotype.*** CN10-O17-NIV11-12, partial skeleton with cranial and postcranial elements.

***Storage.*** IPHES-CERCA facilities.

***Stratigraphic range.***Hitherto restricted to its type locality, Lower Pliocene.

***Diagnosis (genus and species).*** [No trait alone is diagnostic, but the combination of characters is unique within the Talpidae] Small sized mole with dental formula ???3/2143. Doubled mesostyle in M1 and M2. Double rooted P4. Presence of a parastyle in P4. Lower premolar row without gaps. Enlarged i2. Absence of metastylid in m2. Robust and small postcranial remains. Pit for M. flexor digitorum profundus ligament present. Straight medial edge of humeral trochlea. Fusiform shape of the humeral capitulum. Well-developed and transverse olecranon crest. Anconal and coronoid processes present in the ulna. Presence of capitular process in the radius. Scaphoid and lunar not co-ossified.

***Differential diagnosis.*** See Supplementary Information 1. Supplementary Tables of measurements and comparisons with other selected species of Talpidae can be found in Supplementary Information 2 (Tables S1–S9).

### Description

The partial skeleton of the mole lies partially embedded by the sediment exposing the lateral side of the remains. Some skeletal elements are found in anatomical connection such as the mandible, the dentition and the postcranial elements (right forelimb). The tibiofibula is isolated from the rest of elements (Fig. 4).

*Skull and upper dentition.* The skull is partially preserved but strongly damaged. None of the cranial structures can be identified through digital reconstruction. Because the remains are not scattered, the outline can be delineated. The left upper tooth row is incomplete, but it preserves the molars, three premolars and one incisor (Fig. 5a). The incisor is displaced pointing mesially. The length of the premolars decreases towards the anterior part, P4 > P3 > P2. The premolars are all double rooted. P2 (L = 0.80; W = 0.38) is clearly unicuspid. P3 (L = 0.85; W = 0.47) is mostly dominated by a high central cusp with a curved posterior ridge which bears a metastyle at its posterior end. The anterior ridge of both premolars is straight. The protocone in P4 (L = 1.36; W = 1.13) lies mesiolingual to the paracone. The postparacrista is straight and slightly curved at the posterobuccal end. The preparacrista is curved and wide at the end.

The molars are double-rooted and become smaller distally, M1 > M2 > M3. There is no cingulum in any of them. In the M1 (L = 2.27; W = 1.78) the metacone is expanded distolingually. The paracone is slightly larger than the metacone. The trigon basin is deep and wide. The protocone is small in the upper molars (M1 and M2) and the hypocone is also visible in both. The mesostyle is divided into two cusps separated by a deep valley. The parastyle and the metastyle are also well developed. In the M2 (L = 1.82; W = 1.66), the trigon basin forms a deep valley as in M1. The paracone is the highest cusp and the protocone is the smallest. The paracrista is a bit shorter than the postparacrista and similar in length with the postmetacrista. The metastyle and parastyle are well developed. The mesostyle valley follows the same pattern as in M1. In the M3 (L = 1.52; W = 1.35), the trigon basin is wide but not as deep as in the other molars. The metacone and the paracone have a similar height in contrast to the protocone, which is smaller. All the cusps are wide and rounded. The parastyle is well developed.

*Mandible and lower dentition.* Both hemimandibles and most of the lower dentition (Fig. 5b) are preserved. Only the right hemimandible and its whole dentition (Fig. 5c) is exposed but digital reconstructions demonstrate that the left hemimandible additionally preserves two molars, two premolars, the canine and one incisor. The anterior mental foramen is observed below p2 and p3, and a less clear posterior one is placed below m1 and m2.

The corpus mandibulae is slender and elongated. The distal part becomes wider and convex towards the angular process. The anterior profile of the coronoid process is slightly curved and straightens towards the tip of the coronoid process. It has a convex shape with a faint notch. The posterior profile delineates a concave curvature that connects to the condylar process. The condylar process points backwards, and it is longer than the angular process. The angular process is short, and the tip ends anterior to the angular process and posterior to the coronoid process.

Measurements (mm): Length of the corpus mandibulae = 14.63; maximum thickness of mandible (below m2) = 1.40; maximum height of mandible (below m2) = 1.45, minimum thickness of mandible (below i1) = 0.43; Minimum height of mandible (below i2) = 1.12; height of coronoid process = 4.94.

Regarding the dentition, the incisors and the canine present an elongated and flat crown. Both incisors are single-rooted. The i2 is the largest and widest of the incisors. Its shape is spatulated. The canine is smaller than the incisors. The premolars are doubled-rooted unicuspids and there are no gaps between them. The lateral outlines of their crowns are almost triangular. All of them have divergent and rather stout roots with rounded tips. Towards the distal side, premolars are progressively larger, p1 < p2 < p3 < p4. The cusps in all of them are rather high and sharp. The p1 (Right: L = 0.52; W = 0.35; Left: L = 0.50, W = 0.38) stands as a simple single-cusped tooth. The posterior ridge of p2 (Right: L = 0.65; W = 0.33; Left: L = 0.60; W = 0.32) and p3 (L = 0.71; W = 0.33) are curved and wide towards the posterior end. In p4 (L = 0.88; W = 0.59), the anterior ridge of the protoconid is straight. The posterior ridge is slightly convex towards the entoconid.

The molars are doubled-rooted, and they are progressively smaller towards the distal side, m1 > m2 > m3. In the m1 (L = 2.04; W = 1.33), the protoconid is higher than all the lingual cusps, but similar in size to the hypoconid. The talonid basin is delimited anteriorly by the oblique cristid which ends anterolingually between the metaconid and entoconid without the development of a metastylid. The entoconid and metaconid cusps have the same height, being slightly higher than the paraconid. No talonid notch is observed. There is a well-developed entostylid at the distolingual side. In the m2 (Right: L = 1.92; W = 1.32; m2: L = 1.89; W = 1.29), the talonid basin is reduced compared to the m1. The paracristid in m2 is higher than in m1. The oblique cristid follows the same pattern as in m1. The metaconid is higher than the paraconid and slightly higher than the entoconid. Anteriorly, the praecingulid is narrow. The protoconid is higher than the hypoconid. The talonid notch is not observed but the entostylid is well developed as in the m1. The talonid basin in m3 (Right: L = 1.54, W = 1.07; Left: L = 1.53, W = 1.04) is smaller than that of m1 and m2. The metaconid is similar in height to the paraconid but slightly higher than the entoconid. The praecingulid is narrow and the protoconid is higher than the hypoconid as in the m2. The entostylid is not developed.

*Postcranial elements.* The digital reconstructions show a mash of numerous rib fragments and vertebrae flattened by compaction. It has been impossible to restore any element of the postcranial axial skeleton in an objective way. Similarly, one clavicle and scapula are preserved and partially exposed. However, in digital reconstructions they are flattened and strongly fragmented, so it has been impossible to describe the original morphology of any element from the shoulder girdle.

*Humerus.* Only the distal part of the right humerus is preserved (Fig. 6a). At the distal end of the epiphysis, the ectepicondylar (= Lateral epicondyle) and the entepicondylar (= Medial epicondyle) processes are rather well preserved but their tips are missing. In the ectepicondyle, the capitulum is laterally elongated with a fusiform shape. The surface of the entepicondyle is smaller than that of the ectepicondyle. The entepicondylar foramen is shown as a deep groove. Adjacent to this groove, a wide elliptical fossa for *M. flexor digitorum profundus* ligament is observed. At the posterior side of the distal end, the epicondyles are separated by a large trochlear area associated with the broadening of the humerus in which there is a small projection separating the trochlea from the fossa for *M. flexor digitorum profundus* ligament. This area becomes deep towards the shaft of the humerus, giving rise to the olecranon fossa.

Measurements (mm): Length = 7.14; maximum distal width = 6.04; shaft thickness = 2.37; shaft width (minimum diaphysal width) = 3.30.

*Radius.* The right radius (Fig. 6b) is preserved in lateral connection with the distal part of the humerus. At its proximal end, the strong and stout capitular process is followed by the glenoid cavity. The glenoid cavity is deep and concave. In medial view, the ulnar articular facet of the capitular process is elongated and clearly defined. In lateral view, a conspicuous crest extends distally from the capitular process, showing a small groove for the attachment of *abductor pollicis* muscle. In medial view, next to this crest, a wide fossa is observed in which the radial head of abductor muscle is attached. In the distal part of the radius, the scars for tendon of *extensor carpi radialis* muscle forms a slight protuberance between them. The articular facets for the lunar and scaphoid are narrow and convex.

Measurements (mm): Length (Glenoid cavity – articular facet) = 7.56; shaft length = 6.09; proximal width = 2.57; distal width = 2.77; maximum distal thickness = 1.56.

*Ulna.* The right ulna (Fig. 6c) is preserved in connection with the radius and the distal end of the humerus. The proximal crest is elongated in anterior view with a widely extended area of insertion for the triceps. The decrease of the extension of this area towards its distal part forms the medial and lateral olecranon crest, which ends with the protuberance of the anconaeus process. Medial and lateral olecranon crests are elongated increasing the length of the ulna over the radius. In lateral view, a protruding proximal crest is enhanced by the depth of the abductor fossa. Towards its distal part, at the anconaeus-level, this fossa presents a small groove of the abductor scar. Below them, the coronoid process is observed with a small protuberance compared with the anconaeus. This difference in size conforms the semilunar notch. Next to the coronoid process, the radial articular facet overhangs the abductor fossa expanded towards the laterodistal side. In anterior view, the humeral articular facet is observed between the anconaeus and the coronoid process, which forms a slight depression. Below the coronoid process there is a small scar for the insertion of the brachialis muscle. The area delimited by the anconoeus process and the coronoid process is the functional zone for the rotation of the humerus, namely the trochlear area. The distal part of the ulna is wide at the connection of the posterior crest and the abductor fossa with the shaft. In lateral view, the shaft ends with the styloid process, which is large with a stout and rounded terminal process. The ulnar articular facet is narrow and moderately deep ending in a stout cuneiform articular facet.

Measurements (mm): Length = 13.00; olecranon length (proximal crest – anconaeus process = 4.11; mediolateral diameter = 1.30; width of proximal crest = 4.22.

*Carpal bones.* All the carpalia are preserved (Fig. 7a). The triquetrum, hamate, centrale, trapezoid, trapezium and capitate are in connection below the third and fourth metacarpal. The lunate and the scaphoid are disconnected from the hand. In the scaphoid, the groove for the insertion of the *flexor carpi radialis* muscle is observed (Fig. 7b).

*Metacarpal bones.* Four elements of the metacarpalia are preserved, the second (II) being the longest of them (Fig. 7b). The proximal prominence is rounded and stout. The outline of the phalanx articular facet is strong and it forms two protuberances. Generally, the metacarpals sustain the shape of the phalanx articular facet. The size of the metacarpals decreases from II to V. The proximal prominences of the third metacarpal are stouter than that of the second. The metacarpal articular facet is wide. The unciform articular facet is hardly visible. The fifth metacarpal is shorter and narrower than the rest. The unciform articular facet is strong and rounded.

Measurements (mm): Mc II: L = 2.66, W = 1.45, Distal W = 1.63; Mc III: L = 2.31, W = 1.63, Distal W = 1.67; Mc IV: L = 1.57, W = 1.43, Distal W = 1.46; Mc V: L = 1.52, W = 1.37, Distal W = 1.40.

*Phalanges.* Six phalanges are preserved (Fig. 7b). The proximal phalanges are shorter than the metacarpals. They are stout and the proximal articular facet is wide in their connection with the metacarpals. The distal articular facet is wide and rounded to the sides with a similar shape to that of the middle phalanges. The middle phalanges are shorter than the proximal phalanges, and they become narrower towards the distal end. The proximal articular facets are wide and rounded. The distal articular facets are slightly thinner than the proximal ones. Distal phalanges are long, and they flatten towards their distal ends. They have a characteristic shovel-like shape with a small notch at the distal end. The proximal articular facet is narrower than the distal articular facet of the middle phalanges.

Measurements (mm): Pp III: L = 1.44, W = 1.67, Distal W = 1.54 ; Pp IV: L = 1.39, W = 1.46, Distal W = 1.38; Mp III: L = 1.28, W = 1.54, Distal W = 1.01; Mp IV: L = 1.1, W = 1.38, Distal W = 1.06; Dp III: L = 4.28, W = 1.01, Distal W = 0.47; Dp IV: L = 3.60, W = 1.06, Distal W = 0.30.

*Sesamoid bones.* The proximal part of one sesamoid bone is partially preserved (Fig. 7b). The proximal articular facet of this sickle-shaped bone is wide. The distal part is stout. This element has been found below the other bones of the hand. In addition, a small accessory sesamoid bone is observed.

Measurements (mm): L = 3.13; W = 1.28.

*Tibiofibula.* The tibiofibula (Fig. 6d) is placed a few centimeters away from the rest of the connected skeleton of the animal. The tibia is thicker than the fibula and the two bones are separated by the interosseous space. Tibia and fibula are fused at their distal part, thus ending in a wide shaft. In the proximal part, the head of the fibula forms a rounded protuberance. In the tibia, the rounded medial condyle is separated from the lateral condyle by a notch that conforms the intercondyloid fossa. The lateral condyle is longer than the medial one. In the posterior part of this notch, there is a tuberosity which continues towards the distal part of the dorsomedial ridge. This ridge becomes slightly wider mediolaterally at its distal part ending with a slight depression before the medial malleolus protuberance. Next to medial malleolus, in lateral view, a tuberosity conforms the lateral malleolus. In the posterior surface of the distal part, the groove for the *M. flexor digitorum tibialis* is observed. A low narrow ridge of the tibiofibular shaft separates the previous groove from that for the tendon of the *flexor digitorum fibularis* muscle. At the distal end, a short and narrow crest separates the groove for the *flexor digitorum tibialis* from the that for the tibialis posterior tendon. The articular facet is widely extended laterally.

Measurements (mm): Length = 13.97; width = 2.79; width of the distal articular facet = 2.42; interosseous space = 1.68.

*Additional hindlimb bones.* Other than the tibiofibula, one femur, one metatarsal, and one distal phalanx are the only elements of the hindlimb preserved. However, the digital models reconstructed from the micro-CT scans are too flattened and fragmented to be considered a good approach of the original morphologies of these elements. Therefore, these bones do not provide sufficient resolution to be confidently described.

## Discussion

### Systematics and phylogeography

The specimen from CN herein studied is, to our knowledge, the most complete Pliocene talpid skeleton ever reported in Europe. Other similar exceptionally preserved fossils of talpids are the Oligocene specimen of *Geotrypus antiquus* from the German locality of Enspel^26^and the Miocene samples of *Mygalea jaegeri*, *Proscapanus sansaniensis*, and *Geotrypus montisasini* reported by Schwermann and Thompson^16^. Except for these privileged finds, the fossil specimens of the family Talpidae are usually restricted to isolated teeth and some characteristic postcranial elements, which make their correlation difficult. The discovery of the mole in the Pliocene site of CN turned into an exceptional landmark by the combination of unexpected characters in the only specimen recovered.

The European fossil record of fossorial moles during the Pliocene is dominated by *Talpa*^5^. However, some other less frequent talpids have been documented in literature^11–13,17,46,47^. None of the unusual fossil forms described in these works completely fit the morphology or size of the specimen from CN. Indeed, all the Pliocene occurrences of fossorial moles in Spain have been traditionally limited to the species *T. fossilis* and *T. minor*^27^directly ascribing all the wide and robust fossil humeri of talpids to one of these two species^48^. Actually, the specimen CN10-O17-NIV11-12 was tentatively identified as *Talpa minor* at first sight (see Introduction). Subsequent detailed scrutiny of the fossil resulted in the observation of an unusual configuration of the anterior lower toothrow, not typically found in the Talpini, which prompted a thorough phylogenetic analysis (see Material and methods). Together with our new species found, we took the chance to include some other taxa not considered in previous analyses, namely the genera *Myxomygale* (data taken from *M. hutchisoni*, *M. antiqua*) and *Hugueneya* (data taken from *H. primitiva*, and *Hugueneya* sp. in Lopatin^49^and the species *Mongoloscapter zhegalloi*, *Skoczenia copernici*, and *Alpiscaptulus medogensis*).

**Fig. 8:**
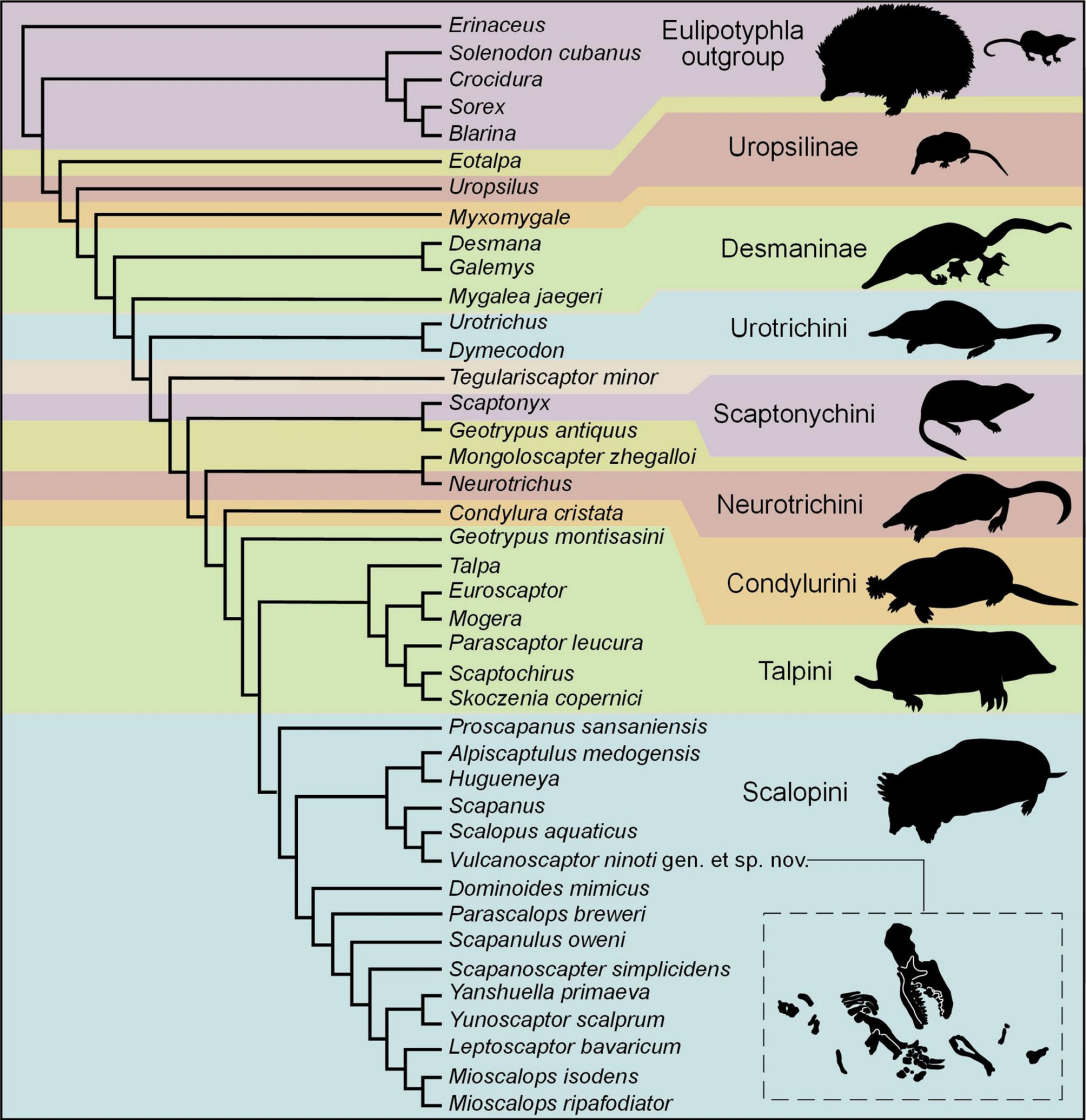
Strict consensus of the most parsimonious phylogenetic tree for the Talpidae (see Material and methods section) including *Vulcanoscaptor* gen. nov. Background colors separate traditional groups, supporting the division into three subfamilies, namely Uropsilinae, Desmaninae, and Talpinae, the latter of which divided into several tribes (Urotrichini, Scaptonichini, Condylurini, Talpini, and Scalopini).

The results of the cladistic analyses performed are shown in Fig. 8. Applying Goloboff’s criterion (K = 2) and equally weighted characters of different types (ordered and unordered) results in the most parsimonious tree possible (CI = 0.3910, CI excluding uninformative characters = 0.3902, HI = 0.7065, HI excluding uninformative characters = 0.6098, RI = 0.6148, RC = 0.2404, G. fit = -97.02592, tree length = 862) (Fig. 8). These results are mostly in line with previous morphological studies^2,3,14,16^ but there are some differences in the resulting evolutionary tree which deserve to be remarked.

Overall, the most evident result is that our evolutionary tree supports the classical morphological view of the family Talpidae divided into three monophyletic clades, namely the subfamilies Uropsilinae, Desmaninae, and Talpinae. Motokawa^50^ reached a similar large-scale picture, getting however different phylogenetic relationships within the Talpinae. The genus *Eotalpa* is placed in a more basal position than Uropsilus (see Hooker^2^). As predicted by Rümke^20^ *Myxomygale* is placed as a more basal taxon than the Desmaninae, clearly separated from the Urotrichini (in which it had been previously included by Ziegler^51^ and Hugueney & Maridet^52^). Desmanines stand as a separate clade from Talpinae in contrast to previous molecular analyses^77^. This situation has been explained by Hooker^2^who suggested separate evolutionary adaptations.The Urotrichini are therefore the most basal forms within the Talpinae. *Tegulariscaptor minor* is closer to the Urotrichini than to the Uropsilinae but it is phylogenetically distinct from *Geotrypus* (stem group to true moles) (see Sansalone et al.^14^). *Geotrypus antiquus* stays separated from *Geotrypus montisasini* (see Schwermann and Thompson^16^), thus leaving *Scaptonyx* as the only member of the Scaptonychini. *Mongoloscapter*, which is included in the Scaptonychini according to Lopatin^53^ is the sister taxon of *Neurotrichus*, a genus that deserved the description of its own tribe [Neurotrichini] according to Hutterer (2005). *Neurotrichus* is therefore separated from *Scaptonyx*, which was formerly considered its sister taxon^50^. The genus *Condylura* stands as the only member of its own tribe [Condylurini], close to the most derived fossorial forms (Talpini and Scalopini). *Skoczenia* is placed in the Talpini tribe, as stated by Rzebik-Kowalska^13^close to *Scaptochirus*. *Parascaptor* is not the sister group of *Scaptochirus* (see Schwermann and Thompson^16^).^14,16^

Within the tribe Scalopini, two main evolutionary lineages are discernible. One of the branches includes the genera *Scapanus* and *Scalopus*, whilst the other includes *Parascalops* and *Scapanulus*. This is in line with the classical view of two subtribes Scalopina and Parascalopina [Hutchison^54^; Schwermann et al^3^]. It is worth of mention that *Alpiscaptulus* and *Hugueneya* are included in the Scalopini (as stated by Chen et al.^4^ and Lopatin^49^ ), but they are clustered with members of the *Scapanus* - *Scalopus* group. The phylogenetic analyses in the original description of *Alpiscaptulus* always placed this genus closer to the *Parascalops* – *Scapanulus* branch. According to our analysis, *Vulcanoscaptor ninoti* gen. et sp. nov., must be also included in the tribe Scalopini, being closely related with *Scalopus* and *Scapanus*. The *Scapanus* – *Scalopus* cluster is a robust group supported by morphological and genetic evidence^3,5,16,50,55,56^). These two extant genera are nowadays restricted to North America, and they are considered ‘the core’ of the subtribe Scalopina. The find of *Vulcanoscaptor* gen. nov. is thus surprising as the only representative of this lineage in Southwestern Europe, and it could raise some questions about its phylogenetic position. Nevertheless, the position of this new taxon in the resulting phylogenetic tree is strongly supported by 11 characters that differentiate it from the rest of the species of the tribe Scalopini, namely: (1) the number of roots of P4 (c. 11); (2) the presence or absence of a paraconule in M2 (c.16); (3) the dimensions of the postmetacrista and preparacrista in M2 (c.20); (4) the presence or absence of a talonid notch in m1-m2 ( c.24); (5) the absence or presence of gaps in the lower premolar row (c.27); (6) the position of the protocone in P4 (c.32); (7) the length of M2 (c.34); (8) the length of M3 (c.35); (9) the length of m1 (c.44); (10) the length of m2 (c.45); and 11) the location of the posterior mental foramen in the lower mandible (c.69).

To find the most parsimonious explanation to the occurrence of *Vulcanoscaptor* in the Pliocene of Europe, there are some observations to be done. First, it is unclear where the origin of the Scalopini should be found. According to Bannikova et al.^55^ the tribe Scalopini first radiated in Eurasia, so the migration of the ancestors of the *Scalopus* – *Scapanus* clade would have reached North America during the Miocene. This hypothesis was claimed to be in line with the observations of Hutchison^54^ who stated that the Scalopini arrived relatively late to that continent, with its oldest occurrences in the Middle Miocene. The phylogenetic relationship of *Scalopus* – *Scapanus* with an Early Pliocene form like *Vulcanoscaptor* is less improbable than initially predicted considering that a relict stock of European Scalopini moles could have survided the Miocene-Pliocene boundary.

A second point refers to the work of Schwermann et al.^3^ who claimed a complex biogeographical history for the Scalopini, with no clear land to place the origin and several transcontinental colonisations. According to their analyses of the fossil record, out-of-North America migrations occurred at least three times during the Miocene. This second option opens the possibility to link our fossil form with the cluster *Scapanus* – *Scalopus* as a relative form who migrated from North America to Europe close to the Miocene – Pliocene transition. Previous works (e.g., Motokawa^50^) had already pointed out the complex evolutionary history of moles, even suggesting that the migrations had North America as destination in four different lineages.

A third consideration is that the uncertainties in the taxonomy of some fossil talpids make difficult to track their paleobiogeographic evolution. The post-Miocene fossil record suggests different biogeographic boundaries or migration routes for the known Scalopini clades depending on the identification of some problematic taxons. For instance, fossil material of *Scapanulus oweni* (the ‘Gansu Mole’) indicate that this species has been present in China at least since Middle Pleistocene times^57^. The Pliocene species from Poland formerly known as *Scapanulus agrarius* described by Skoczen^46^ would be indicative of a European origin for the genus and a Eurasian distribution. However, this species is currently considered a junior homonym of *Parascalops fossilis* (see Rzebik-Kowalska^13^). The only living species of this genus is *P. breweri*, occurring in the northeast of United States and the southeast of Canada. In North America the oldest fossils of *Parascalops* are of Pliocene age Oberg and Samuels^58^ thus completely redrawing the paleobiogeographic picture on the evolution of the subtribe Parascalopina. This is to be added to the systematic uncertainties of some other fossil taxa, such as *Yanshuella* and *Yunoscaptor*, which make unclear how many times these moles have moved from one to another continent^3^.

In any case, our results strongly support the nature of *Vulcanoscaptor ninoti* gen. et sp. nov. as a new Scalopini. In this sense, the phylogenetic analysis carried out is reinforcing the results previously reached by Barrow & MacLeod^59^ on the morphology of talpid mandibles or on the humeri^60,61^. Nevertheless, this Scalopini form is clearly different from any other genus in the tribe because many of them do not share the same dental formula and / or they display different configurations of their postcranial bones. More specifically, the humerus of our mole resembles that of *Condylura* in gross proportions, but the distal part acquires an intermediate shape between *Parascalops* and *Scapanus*, with a notch in the trochlear area less pronounced than in *Parascalops* (also than in *Mioscalops* and *Scapanulus*), but more evident than in *Scapanus*. Moreover, the lateral and medial epicondyles of *Vulcanoscaptor* gen. nov. are more robust than in *Parascalops*, but weaker than in *Scapanus*. Similarly, the proximal end of the ulna of *Vulcanoscaptor ninoti* gen. et sp. nov. is similar to *Mioscalops* in the broad area for the insertion of the triceps muscle. This morphology is also similar to that of *Condylura* (see Hutchinson^54^), with the exception of the olecranon process and the articular facets, which show an intermediate morphology between *Parascalops* and *Scapanus*.

### Paleobiology and taphonomy

Adaptations to subterranean environments imply a high development of the humerus and a subsequent impact on other functional traits, in which the anterior mobility of the forelimb is affected by maximizing the abduction movement^62^. According to Meier et al.^60^such modifications are clearly reflected in an extremely short, broad and compact bone specialized for digging. The complexity of the humerus is the result from the high load that the moles must overcome with the forelimbs when digging. Supporting such intense mechanical strains requires a great development of the muscles involved, mainly those of the triceps muscular complex and their attachments sites^60,62,63^. The dimensions and the compaction of the humerus herein described, together with the strong development of the forearm, imply a huge development of the abductor muscles (i.e. *teres major*, *pectoralis*, *subscapularis* and *latissimus dorsi*). According to Gambaryan et al.^64^ these traits are related with a major stabilization of the forearm at the elbow and wrist joints in order to maintain the humerus and the hand in the correct position for the lateral thrust. Meier et al.^60^ detailed that in the fossorial clades (i.e., Talpini and Scalopini) the humeri showed a rather buckled outline and a slightly elliptic medullary cavity, reflecting the torsion of this element and the deep reaching distal end of the deltopectoral crest. Therefore, there is no doubt that *Vulcanoscaptor ninoti* gen. nov. et sp. was a highly specialized burrower (for further details, see extended discussion in Supplementary Information 3).

The find in lacustrine sediments of a mole highly specialized in burrowing deserves some comments. It can be speculated that the presence of this specimen in the anoxic bottom of the lake could be related to a possible semi-aquatic practice other than strictly burrowing. It would be difficult to assess this from the scarce record found at CN and more evidence and analysis are needed to sustain this hypothesis. However, several authors have documented swimming abilities among talpids adapted to strictly fossorial lifestyles (e.g., Hickman^65^ and references therein). The same adaptation of the humerus needed for digging could also be useful for swimming^66^. Moreover, other features described are comparable with the aquatic *Condylura.*

The presence of a mole in the sediments of a lake suggest that this individual should have lived in the nearby area. It could have been dragged into the lake by a predator, like a bird^67^ or by floods, or accidentally fell and drowned.

The specimen here described is incomplete, and semi-articulated as it preserves a good anatomical connection among several skeletal elements but not within each portion (e.g. cervical vertebras). Dispersal of the skeletal elements could have occurred after or before deposition of the remains at the anoxic bottom of the lake as suggested by the missing anatomical portions^26,67–70^. A rapid burial in the ground or in deep waters^71^instead, would have preserved most of the anatomical connections by impeding refloat to happen after developing decomposition gasses. Moreover, the anoxic bottom of the lake also prevents major bioturbators to disturb the carcass. Complete articulated body are common in maar sites^72^ including CN^31,73^. However, this mole specimen represents an exception. The lateral position could suggest that it arrived to the final deposition site already incomplete, as similar situations suggest that moles will lay ventrally or dorsally, but not laterally due to the shoulder girdle^26,67^. This specimen could represent the remains of a scavenged carcass accidentally fallen in the lake or a floated carcass that sank after decomposition gases escaped. Lastly, post depositional processes such as faulting that affected CN could also be responsible for the differential preservation of some elements^74,75^. For further details, see extended discussion in Supplementary Information 3.

## Conclusions

The discovery of the partial skeleton of a mole in CN enlarges the fossil record of small mammals from this locality. Comparisons of our specimen with the Pliocene and extant talpids result in its identification as a new genus and species included in the tribe Scalopini by an unusual combination of morphological characters of its dentition and postcranial elements. This is an unexpected find, considering that Scalopini moles were not frequent in Europe after the Miocene.

The phylogenetic analysis performed to place this new find has resulted in a major consensus tree, which fits quite well the models based on morphological characters previously obtained by other authors. In this sense, only a few new locations of some taxa within the phylogeny of talpids are discerned. The inclusion of the characters codified for *Myxomygale*, *Hugueneya*, *Mongoloscapter*, *Skoczenia*, and *Alpiscaptulus*, together with the new ones of *Vulcanoscaptor* gen. nov. has resulted in new positions for the genera *Eotalpa*, *Tegulariscaptor*, *Geotrypus*, and *Neurotrichus*.

With respect to its adaptations, digital reconstructions display several traits of the forelimb which implies intense modifications of the forearm as a respond of the biomechanics of the humerus to achieve great efficiency throughout digging. This is clearly evidencing that *Vulcanoscaptor ninoti* nov. gen et sp. was adapted to a fossorial lifestyle.

The strange occurrence of this mole in the fossil site of CN is a difficult issue to tackle for which different scenarios are suggested. The disposition and the optimal preservation of the partial skeleton of the mole indicates that whatever the cause of death, the specimen probably deposited already partially disarticulated. Posteriorly, the soft tissues were decomposed promoting the disarticulation of other remains before their compaction and fragmentation.

The capability of the mole for swimming whereby it would have reached the lake deliberately, or the absence of this quality, could determine whether the mole died on the shore during a regular soak or drowned after falling into the lake. The real causes of death and how the specimen arrived at lacustrine sediments remain uncertain and they will deserve further research.

## Materials and methods

The great extension of the excavation area (approximately 275,000 m^2^) required a complex level of organization, divided into several sectors, pits, sections, layers, units and subunits. Up to date, five sectors have been excavated: Can Argilera, Can Pol, Can Cateura, Comercial and Butano sectors. To define the limits of the lake and the fossiliferous layers, several pits of about 4–5 m of maximum depth were dug out with a backhoe. Their dimensions varied depending on the evidence of the potential fossil content. Subsequently, the fossil strata were manually excavated. Reference systems such as UTM (ETRS89) and Cartesian coordinates were used to position the pits and fossil occurrences. The placement of the fossil was recorded by means of a registration code (acronym of the site -CN-, year, sector, pit, excavation unit, square and number of register). Depending on the preservation of the remains and the materials in which they were included, different methods were applied to unearth them^31^.

The specimen studied in the present work was found in the layer 11 of the subunit 2.3 (Fig. 3). In terms of registration, this subunit belongs to the pit 7/8 from the Can Argilera sector. The remains were preserved at the surface of green clayish sediments with fine-grained sandy admixture. The specimen was originally extracted in its matrix from the excavation with expanded polyurethane. After extraction, the remains were taken to the IPHES-CERCA laboratory for preparation. Once there, its excavation was completed using mechanical methods and the aid of a binocular lens.

After delimiting the skeletal remains, the clay block and the specimen were consolidated with ethyl silicate (Estel 1000), which was drip-applied using a syringe onto the surface of the block and the skeleton. In this case, the specimen was consolidated in several sessions without saturating the sample to avoid an excess of siliceous crystallizations on the surface. Once hardened, the block was reduced and prepared for Computerized Microtomography dividing it in five fragments. Subsequently, the fragments were adhered with acrylic resin, Paraloid^®^ B-72 dissolved in 20% acetone. Finally, a structural reintegration was carried out with a putty made from the sediment of the same block agglutinated with the same acrylic resin used for adhesion. For storage, a custom-made polyethylene-based support was made in different formats: Ethafoam^®^ foam, Tivek^®^ tissue and a bag.

Because the remains are partially embedded, the morphological description of this mole for subsequent taxonomic identification is complicated. To avoid the extraction of the fossil, the virtual reconstruction of the remains was carried out using computerized tomography, a nondestructive technique. Five µ-CT scanners, with a 3D spatial resolution of up to 6 μm, were made with the V|Tome|X s 240 (GE Sensing & Inspections Technologies) at the CENIEH in Burgos (Spain). The set of images obtained after the scans were processed with the free and open access software ‘3D slicer’. Subsequently, and based on these models, the descriptions and measurements were made according to Hutchison^7,54^.

*Cladistic analysis -* A total of 41 extant and extinct species have been scored based on morphological characters, both cranial and postcranial elements (Supplementary Information 4). The list of characters and taxa suggested for phylogenetic analyses was carried out based on that proposed by Schwermann et al.^3^ and its preceding works^2,16,66^. When some specific information was missing, the data matrix was completed checking the characters of selected specimens of some subfamilies, tribes, genera and species (Supplementary Information 4). For details of the different species and the skeletal remains see Schwermann et al.^3^.

A total of 175 characters have been considered to score the different taxa based on dentition, humerus, hand and tibiofibula. Of these discrete characters 114 are binary and 61 are multistates. Some taxa (e.g., *Mioscalops isodens*, *Domninoides mimicus*, *Parascalops breweri*) show polymorphism for some characters which are named as letters codifying different states (Supplementary Information 4). In view of the absence of preserved skeletal elements of some species, characters have been scored as missing with the symbol “?“. Finally, in the present work we have added some taxa to the list: (1) *Tegulariscaptor minor* from Sansalone^14^; (2) *Myxomygale hutchisoni* sensu Klietmann et al.^76^. and *Myxomygale antiqua* sensu Hugueney & Maridet^52^; (3) *Mongoloscapter zhegalloi* according to Lopatin^53^; (4) *Skoczenia copernici* sensu Rzebik-Kowalska^13^ *Alpiscaptulus medogensis* by Chen et al.^4^; (5) *Hugueneya* sp. in Lopatin^49^ and *Hugueneya primitiva* according to Van den Hoek Ostende^77^ and (6) the new taxon herein described, *Vulcanoscaptor ninoti* gen. et sp. nov.

For cladistic analyses the software PAUP 4.0^78^ has been used to obtain the most parsimonious tree possible. The parsimony criteria have been applied by means of a heuristic search. For further veracity of the obtained tree, Bremer values^79^ were calculated in addition to the application of the scheme of implied weights from Goloboff^80^. For the latter criterion, a value of K = 2 was applied to reduce the homoplasy. On the other hand, when setting character types, the criterion of equal weights and a status as unordered or non-additive has been applied to all characters. Exceptionally, 27 of those characters have been considered as ordered or additive based on Wagner’s parsimony criterion due to the assumption of an ordered sequence according to their position in the symbol list^81^. Finally, in our analyses *Erinaceus europaeus* is considered as an outgroup following different works^2,16,66^.

*Anatomical abbreviations* – **abf**: abductor fossa, **abs**: abductor scar, **acs**: accessory sesamoid bone **agp**: angular process, **anc**: ancanoeus process, **brs**: brachialis scar, **c**: oblique cristid, **caf**: cuneiform articular facet, **cdp**: condylar process, **cpp**: capitular process, **cpt**: *capitulum*, **crp**: coronoid process, **dmr**: dorso medial ridge, **dp**: distal phalanx, **ef**: entepicondylar foramen, **end**: entoconid, **fdf**: *flexor digitorium fibularis* tendon, **fdt**: *flexor digitorium tibialis*, **ffd**: fossa of *m. flexor digitorium* ligament, **flc**: falciform, **ga**: groove for *abductor pollicis longus* tendon, **hf**: head of the fibula, **hff**: humeral articular facet, **hyd**: hypoconid, **hyl**: hypoconulid, **i**: incisor, **ins**: interosseus space, **le**: lateral epicondyle, **llc**: lateral olecranon, **lm**: lateral malleolus, **lrf**: lunar articular facet, **ltc**: lateral condyle, **m**: molar, **mc**: metacarpal, **md**: medial phalanx, **mdc**: medial condyle, **me**: medial epicondyle, **me**: metacone, **med**: metaconid, **mes**: mesostyle, **met**: metastyle, **mld**: medial olecranon, **mm**: medial malleolus, **of**: olecranon fossa, **p**: premolar, p.a.: paracone, **pad**: paraconid, **par**: parastyle, **pc**: pectoral crest, **pp**: proximal phalanx, **pr**: protocone, **prc**: praecingulid, **prd**: protoconid, **prt**: protoconule, **pxc**: proximal crest, **raf**: fossa for radial head of m. abductor, **rf**: radial articular facet, **scp**: scaphoide, **sf**: scaphoid articular facet, **sn**: semilunar notch, **sty**: styloid process, **ta**: trochlear area, **tmp**: terminal process, **tp**: tibialis posterior tendon, **ulf**: ulnar articular facet.

*Institutional abbreviations* – CENIEH (Centro Nacional de Investigación sobre la Evolución Humana, Burgos, Spain); IPHES (Institut de Paleoecologia Humana i Evolució Social, Tarragona, Spain).

## Electronic supplementary material

Below is the link to the electronic supplementary material.

Supplementary Material 1

Supplementary Material 2

Supplementary Material 3

Supplementary Material 4

## Data Availability

Holotype: The fossil elements of *Vulcanoscaptor ninoti* gen. et sp. nov. are hosted in the IPHES-CERCA with the reference: CN’10. Can Argilera sector. Pit 7/8. Layers 11. Square O17. Number 12. The 3D virtual models of *Vulcanoscaptor ninoti* gen. et sp. nov. are accessible for viewing in the open-source 3D repository Morphosource (https://doi.org/10.17602/M2/M614677; https://doi.org/10.17602/M2/M614683; https://doi.org/10.17602/M2/M609966).
