# Supplementary material for: An unexpected Scalopini mole (Talpidae, Mammalia) from the Pliocene of Europe sheds light on the phylogeny of talpids
Source: Sci Rep. 2025 Jul 10;15:24928. doi: 10.1038/s41598-025-10396-1 (PMC12246231; doi:10.1038/s41598-025-10396-1)
Supplement: Supplementary file 1 — Supplementary Material 1 [file 41598_2025_10396_MOESM1_ESM.docx]

Supplementary Information 3 for “An unexpected Scalopini mole (Talpidae, Mammalia) from the Pliocene of Europe sheds light on the phylogeny of talpids”.

Adriana Linares-Martín, Marc Furió, Bruno Gómez de Soler, Jordi Agustí, Oriol Oms, Federica Grandi, Hugues-Alexandre Blain, Elena Moreno-Ribas, Pedro Piñero, Gerard Campeny

Paleobiology

Throughout the history of the talpid lineage, the humerus has been the bone which better has reflected the evolution of this family, especially in fossorial moles. Adaptation efforts in subterranean environments imply a greater development of this bone and a subsequent impact on other functional traits, so the anterior mobility of the forelimb is affected by maximizing the abduction movement ^1^. According to Meier *et al.* ^2^, such modifications are clearly reflected in an extremely short, broad and compact bone specialized for digging. The complexity of the humerus is the result from the high load that the moles must overcome with the forelimbs when digging. Supporting such intense mechanical strains need from a great development of the muscles involved, mainly those of the triceps muscular complex and their attachments sites ^1,2,3^.

In the present case, the humerus displayed by the digital models is small and broad despite of the proximal part is hard to define. The optimal conservation of the distal epiphysis and the strong development of its morphology allows to identify different features related with a fossorial lifestyle. Furthermore, the compactness of this bone in the Scalopini is verified in Meier *et al.* ^2^ and associated with fully digging adaptations.

The sequence of movements associated to the kinematics of digging is closely related with the biomechanics of the humerus (excellently detailed in Gambaryan *et al.* ^4^). Before the first movement of digging, the mole shifts the hand to the head placing it in the front of the muzzle. This implies a hyperextension of the shoulder joint and a hyperabduction of the humerus in which the distal end of this bone is directed forward and upward with respect to the shoulder joint. The following movement, the abduction of the humerus, place the hand in the correct position to generate a laterally directed forced on the substrate. To achieve a high performance and decrease the stress and the humeral mobility during these movements, the loadings are distributed in well-developed muscles such as the *flexor digitorum profundus*, the pronator teres, the extensors and the abductors ^1^.

During the humerus abduction, the pressure of the hand to move away the soil must be high, increasing the strain of the *flexor digitorum profundus* ligament. To achieve this, Gambaryan *et al.* ^4^ suggested that the moment of maximum force of the abductor muscles is attained when the plane of the humerus is perpendicular to the parasagittal plane. The point is keeping the humerus in a limited rotation while its plane is perpendicular. This is accompanied with the shoulder and elbow joints which must be flexed in order to reduce the arm length while increases the force of the hand in the soil. This mechanism constrains the rotation of the humerus pronation in which the medial epicondyle is placed caudolaterally and the lateral craniomedially. At the same time, extension-flexion axis of the hand must be in parasagittal position. When the forces decrease, at the end of the lateral thrust, the humerus is placed in supination.

This complex mechanism is reflected in the morphologies of the arm bones of the mole herein described. In the distal part of the humerus, the lateral epicondyle is strongly developed for the extensor muscles (i.e. *triceps caput longum*, *triceps caput laterale*, *anconeus lateralis*, *anconeus medialis*) and the fossa for the insertion of the ligament of the *flexor digitorum profundus* muscle is deep and wide. This is indicative of a strong ligament and a powerful muscle. In the same way, the capitulum forms a huge and rounded protuberance and the trochlear area ⸺where the ulna fits⸺ is broad. Actually, the proximal part of the ulna and the radius are also strongly modified. Therefore, the muscles involved as the pronator teres and the extensors must have been strong. At the distal part, the articular facets of the ulna and radius are convex and narrow.

The high degree of fragmentation of the possible scapula and the damage at the proximal part of the humerus do not provide additional information in this issue. Nevertheless, the dimensions and the compaction of the humerus herein described, together with the strong development of the forearm, imply a huge development of the abductor muscles (i.e. *teres major*, *pectoralis*, *subscapularis* and *latissimus dorsi*). These traits are related with a major stabilization of the forearm at the elbow and wrist joints in order to maintain the humerus and the hand in the correct position for the lateral thrust ^4^.

Lastly, the huge development of the antebrachium is reflected in the manus. Sánchez-Villagra and Menke ^5^ suggested that the presence of a small sesamoid element without evidence of articular facets and attached to the triquetrum by connective tissue is related with an ulno-palmar extension and a powerful shovel. In the specimen herein studied, this bone is preserved disconnected because of the loss of the soft tissue. However, this sesamoid element together with the enlargement of the triquetrum and the presence of the falciform is likely associated with a broad shovel.

In terms of force, the degree of development of the muscles involved transcend the purpose of this paper but there is no doubt that it presents an optimized mechanism for burrowing. Besides the huge compactness of the humerus, Meier *et al.* ^2^ detailed that in the fossorial clades (i.e., Talpini and Scalopini) the humeri showed a rather buckled outline and a slightly elliptic medullary cavity, reflecting the torsion of this element and the deep reaching distal end of the deltopectoral crest. Therefore, there is no doubt that *Vulcanoscaptor ninoti* gen. nov. et sp. was a highly specialized burrower.

Taphonomy

Several articulated, semi-articulated and disarticulated vertebrates have been recovered at Camp dels Ninots (CN) fossil Lagerstätte ^6-9^. Most of the specimens recovered belong to layer 11, a deposition of greenish silts and clays rich in diatomites. These have been collected in different outcrops, indicating that accumulation processes in this unit could be a repeated phenomenon through time and space. The preservation of large and small vertebrate skeletons in layer 11 is usually in good anatomical connection ^6,9,10^, but posterior depositional processes such as faulting of the site ^11^ participated in structural damage of some elements ^8^.

The partial skeleton herein studied has been found in semi anatomical connection and lying laterally. This arrangement is commonly found in most fossil-Lagerstätte ^12,13^, except when the anatomy of the animal does not allow it ^14,15^. Specifically, for the case of the mole from Enspel fossil-Lagerstätte ^16^, the authors suggest that this specimen lies on the ventral side most probably due to the anatomy of the shoulder girdle. The preserved portion is almost complete with elements from both the right and left side, however, the mole specimen from CN only has one side represented. Several skeletal parts, such as the skull, mandibles and some postcranial elements are in anatomical connection but there is some degree of disarticulation in the peripheries of the carcass. For instance, some bones of the hindlimb and the manus are displaced from the rest of the elements. Digital reconstructions show an intensive fragmentation of the skull and thoracic elements such as ribs, vertebrae or the scapula, which are embedded in the sediment, but no other skeletal elements were found with the CT scan.

In similar situations ^16-18^ the incomplete nature of the specimens the authors are discussing is due to the limit of the slab, implying that the remaining body was not recovered or was damaged when the specimen was collected. The CN mole was excavated *in situ* and the area surrounding the finding have been searched for. It is still possible that some small elements could have been missed but it is highly probable that the almost totality of the specimen have been recovered.

Differential preservation of some skeletal element is also taken into account, specifically considering the nature of the CN deposit. faulting and fissuring of the deposit are known processes, causing misalignment of the original horizontal deposition ^11^, as also seen in Figure 4. We cannot exclude that such small elements could have been selectively damaged or moved by these processes.

Dispersal of skeletal elements could be due to different factors such as scavenging, water currents, plant growth or soil erosion and secondary deposition ^19^. The latter case has been suggested for the accumulated fauna encountered in the Pliocene Pula Maar ^20,21^. However, geological characteristic of the CN deposit suggests a relatively calm and anoxic environment for layer 11, with average low sedimentary rate of 0.19 mm·year−1 ^22,23^, which probably allowed a slow decomposition of the soft tissues. The absence of bioturbation and necrophagous activities can be confidently explained by anoxic waters. However, the presence of some fish species such as *Barbus* (‘*Luciobarbus*’) and *Leuciscus* suggests that the anoxia was only occasional, seasonal, or not developed in all parts of the maar ^10^.

Additionally, dispersal of skeletal elements in aquatic contexts could also be due to bloating and floating of the carcasses ^14,19,24,25^. The decomposing gasses developed in the abdominal cavities of the carcass would keep the body afloat until its bursting, leading to a subsequent sinking into the anoxic bottom. In the experimental case discussed by Syme and Salisbury ^26^, the floating carcasses lost the anatomical connection when touching the ground and skeletal elements are found scattered around the body. A rapid burial, instead, would have preserved most of the anatomical connections by impeding refloat to occur ^14,15^. In alternative, the pressure from the water column could also neglect refloating ^18,25^.

Usually, specimens at CN present higher representation of anatomical portions, making the case of the *Vulcanoscaptor ninoti* gen. nov. et sp. different from the others. The find in lacustrine sediments of a mole highly specialized in burrowing deserves some comments.

The mole could have reached the lake deliberately due to a semi-aquatic practice other than strictly burrowing. Such capabilities would be difficult to deduce from the simple study of skeletal morphology and the biomechanics, but several authors have documented swimming abilities among talpids adapted to strictly fossorial lifestyles (e.g., Hickman ^27^, and references therein). In fact, Sánchez-Villagra *et al.* ^28^ pointed that the humeral propulsion for swimming presents similar selective forces to digging. Actually, *Scalopus aquaticus*, a burrowing species, does swim well propelling itself with the webbed hindfeet ^29^. Furthermore, several features of the manus of *V. ninoti* gen. nov. et sp. are comparable with those showed in *Condylura* such as the presence of a sesamoid bone, the not-fused scaphoid and lunate bones and the ulno-palmar extension^5^. Sansalone *et al.* ^30^ also compared the wide distal region of the humerus of a fossorial mole with that shown in *Condylura*. According to the traits of the bones of the forelimb described in this manuscript there could be a relation with the humerus and the manus of the aquatic *Condylura*. As such, the fossilization of *Vulcanoscaptor* could be an accidental event indicative of accessorial swimming abilities of this species.

It may also be the case that the mole simply fell into the lake in an unfortunate accident and drowned after the fur got saturated with water and sank the animal as suggested by Mähler *et al.*^18^. Also, the mole could have been dragged by a bird and fallen into the lake after the bird lost its prey ^18^.

Indeed, talpids are constrained by the degree of humidity and tend to move near the fluvial system, but during arid periods they can occupy soils partially flooded ^31,32^. As the associated fauna in layer 11 (*e.g*., tapirs, turtles, frogs) is likely indicative of a humid period ^22^, the mole could have been dragged alive into the lake by some kind of flooding with no chances of escaping to a safer place. It could have also died near the shore whether by natural death or other external factors. Whatever the cause of death, for the corpse to remain intact and in anatomical connection, a rapid burial or descending to anoxic bottom had to occur early after death.

The mole specimen of CN does not present these characteristics. Even considering post-depositional processes causing the dispersal of some skeletal elements like the case of the mole at Enspel ^16^, they cannot explain how the left side is completely missing. On the contrary, the whole weight of the carcass would have prevented the left side from moving until advanced decay happened. Moreover, the adhesion effect sometimes occurring in lacustrine and marine settings ^33^ would have stick the left side on the ground. While the experimental study of Mähler *et al.* ^18^ demonstrated that moles could lie ventrally or dorsally at the bottom, the fact that the CN specimen lies on the lateral side, strongly suggest that these remains arrived already partially disarticulated at the deposition spot.

It is also possible that the sub-aquatic plants, preserved in the lacustrine sediments of CN, could have trapped the corpse and differential disarticulation ^16,34,35^, caused the detachment of some portions before others, and currents could have moved the remains further away. The disposition of the skeletal elements of the hindlimb (i.e. tibiofibula and femur) in the proximity of the carcass could corroborate this.

In any case, further evidence or analysis on this same individual or other fossils from the same site will be required to strengthen these hypotheses (or arise an alternative one) on how this mole reached the bottom of the lake. In the same way, more postcranial remains from *Vulcanoscaptor ninoti* gen. nov. et sp. would be necessary to support the hypothesis of the semi-aquatic and burrowing lifestyle of this species.

References for Supplementary Information 3

(1) Sansalone, G. *et al.* Decoupling Functional and Morphological Convergence, the Study Case of Fossorial Mammalia. *Front Earth Sci (Lausanne)* **8**, 112 (2020).

(2) Meier, P. S., Bickelmann, C., Scheyer, T. M., Koyabu, D. & Sánchez-Villagra, M. R. Evolution of bone compactness in extant and extinct moles (Talpidae): Exploring humeral microstructure in small fossorial mammals. *BMC Evol Biol* **13**, (2013).

(3) Furió, M. The shrew pleads ‘not guilty’ to the mole’s murder: comment on Bennàsar et al. (2015). *Hist Biol* **29**, 230-233. <https://doi.org/10.1080/08912963.2016.1151016> (2017).

(4) Gambaryan, P. P., Gasc, J.-P. & Renous, S. Cinefluorographical study of the burrowing movements in the common mole, Talpa europaea (Lipotyphla, Talpidae). *Russ J Theriol* **1**, 91–109 (2002).

(5) Sánchez-Villagra, M.R., Menke, P.R., 2005. The mole’s thumb – evolution of the hand skeleton in talpids (Mammalia). Zoology, 108, 3–12.

(6) Gómez de Soler, B. *et al.* A new key locality for the Pliocene vertebrate record of Europe: The Camp dels Ninots maar (NE Spain). *Geologica Acta* (2012) doi:10.1344/105.000001702.

(7) Pandolfi, L., Sorbelli, L., Oms, O., Rodriguez-Salgado, P., Campeny, G., de Soler, B. G., … Madurell-Malapeira, J. (2023). The *Tapirus* from Camp dels Ninots (NE Iberia): implications for morphology, morphometry and phylogeny of Neogene Tapiridae. *Journal of Systematic Palaeontology*, *21*(1). https://doi.org/10.1080/14772019.2023.2250117

(8) Grandi, F., Del Valle, H., Cáceres, I., Rodríguez-Salgado, P., Oms, O., Fernández-Jalvo, Y., García, F., Campeny, G., & Gomez de Soler, B. (2023). Exceptional preservation of large fossil vertebrates in a volcanic setting (Camp dels Ninots, Spain). Historical Biology, 35(7), 1234-1249.

(9) Blain, H. A., Přikryl, T., Cáceres, I., Rodríguez-Salgado, P., Martínez-Monzón, A., Linares-Martín, A., Lozano-Fernández, I., Moreno-Ribas, E., Grandi, F., Oms, O., Agustí, J., Campeny Vall-Llosera, G. & Gómez de Soler, B. (2024). Skeletal taphonomy of the water frogs (Amphibia: Anura) from the Pit 7/8 of the Pliocene Camp dels Ninots site (Caldes de Malavella, NE Spain). Historical Biology, 36(9), 1951-1978.

(10) Přikryl, T. *et al.* Fish fauna of the Camp dels Ninots locality (Pliocene; Caldes de Malavella, province of Girona, Spain) – first results with notes on palaeoecology and taphonomy. *Hist Biol* **28**, 347–357. <https://doi.org/10.1080/08912963.2014.934820> (2016).

(11) Bolós, X., Oms, O., Rodríguez-Salgado, P., Martí, J., Gómez De Soler, B., Campeny, G., 2021. Eruptive evolution and 3D geological modeling of Camp dels Ninots maar-diatreme (Catalonia) through continuous intra-crater drill coring. J. Volcanol. Geotherm. Res. 419, 107369. <https://doi.org/10.1016/j.jvolgeores.2021.107369>

(12) Métais G, Sen S (2018) The late Miocene mammals from the Konservat-Lagerstätte of Saint-Bauzile (Ardèche, France). Comptes Rendus Palevol. 17(7):479–493. https://doi.org/10.1016/j.crpv.2018.05.001

(13) Uhl, D., Wuttke, M., Aiglstorfer, M., Gee, C.T., Grandi, F., Höltke, O., Kaiser, T.M., Kaulfuss, U., Lee, D., Lehmann, T., Oms, O., Poschmann, M.J., Rasser, M.J., Schindler, T., Smith, K.T., Suhr, P., Wappler, T., Wedmann, S., 2024. Deep‑time maar lakes and other volcanogenic lakes as Fossil‑Lagerstatten – An overview. Palaeobiodiversity and Palaeoenvironments (2024) 104:763–848 DOI: https://doi.org/10.1007/s12549-024-00635-0

(14) Beardmore, S.R., Orr, P.J., Manzocchi, T., Furrer, H., Johnson, C., 2012. Death, decay and disarticulation: Modelling the skeletal taphonomy of marine reptiles demonstrated using Serpianosaurus (Reptilia; Sauropterygia). Palaeogeography, Palaeoclimatology, Palaeoecology 337–338 (2012) 1–13. DOI: 10.1016/j.palaeo.2012.03.018

(15) Esperante, R., Brand, L.R., Chadwick, A.V., Poma, O., 2015. Taphonomy and paleoenvironmental conditions of deposition of fossil whales in the diatomaceous sediments of the Miocene/Pliocene Pisco Formation, southern Peru—A new fossil-lagerstätte. Palaeogeography, Palaeoclimatology, Palaeoecology 417 (2015) 337–370. DOI: <http://dx.doi.org/10.1016/j.palaeo.2014.09.029>

(16) Schwermann, A. H. & Martin, T. A partial skeleton of Geotrypus antiquus (Talpidae, Mammalia) from the Late Oligocene of the Enspel fossillagerstätte in Germany. *Palaontol Z* **86**, 409–439 (2012).

(17) Engler T, Martin T (2015) A partial skeleton of the eomyid Eomyodon volkeri Engesser, 1987 (Mammalia: Rodentia) from the late Oligocene Fossil-Lagerstätte of Enspel, Germany. Palaeobio Palaeoenv. 95(1):133–147. http://doi.org/10.1007/s12549-014-0174-8

(18) Mähler, B., Schwermann, A. H., Wuttke, M., Schultz, J. A. & Martin, T. Four-dimensional virtopsy and the taphonomy of a mole from the Oligocene of Lake Enspel (Germany). *Paleobiodivers Paleoenviron* **95**, 115–131 (2015).

(19) Behrensmeyer, A.K., 1991. Terrestrial vertebrate accumulations. In: Allison, P.A., Briggs, D.E.G. (Eds.), Taphonomy: Releasing the Data Locked in the Fossil Record. Plenum Press, New York, pp. 291–335

(20) Sabol, M. *et al.* Early Late Pliocene site of Hajnáčka I (Southern Slovakia) - Geology, palaeovolcanic evolution, fossil assemblages and palaeoenvironment. *CFS Courier Forschungsinstitut Senckenberg* 261–274 (2006).

(21) Kovács J, Németh K, Szabó P, Kocsis L, Kereszturi G, Újvári G, Vennemann T (2020) Volcanism and paleoenvironment of the pula maar complex: A pliocene terrestrial fossil site in Central Europe (Hungary). Palaeogeogr Palaeoclimatol Palaeoecol. 537: 15. https://doi.org/10.1016/j.palaeo.2019.109398

(22) Jiménez-Moreno, G. *et al.* Late Pliocene vegetation and orbital-scale climate changes from the western Mediterranean area. *Glob Planet Change* **108**, 15–28. <https://doi.org/10.1016/j.gloplacha.2013.05.012> (2013).

(23) Rodríguez-Salgado, P. *et al.* Mineralogical proxies of a Pliocene maar lake recording changes in precipitation at the Camp dels Ninots (Pliocene, NE Iberia). *Sediment Geol* **418**, 105910. <https://doi.org/10.1016/j.sedgeo.2021.105910> (2021).

(24) Reisdorf, A.G., Bux, R., Wyler, D., Benecke, M., Klug, C., Maisch, M.W., Fornaro, P., Wetzel, A., 2012. Float, explode or sink: postmortem fate of lung-breathing marine vertebrates. Palaeobio Palaeoenv (2012) 92:67–81. DOI: 10.1007/s12549-011-0067-z

(25) Moore, M., J., Mitchell, G. H., Rowles, T., K., Early, G., 2020. Dead Cetacean? Beach, Bloat, Float, Sink. Front. Mar. Sci. 7:333. DOI: 10.3389/fmars.2020.00333

(26) Syme, C.E., Salisbury, S.W., 2014. Patterns of aquatic decay and disarticulation in juvenile Indo-Pacific crocodiles (Crocodylus porosus), and implications for the taphonomic interpretation of fossil crocodyliform material. Palaeogeography, Palaeoclimatology, Palaeoecology 412 (2014) 108–123. <http://dx.doi.org/10.1016/j.palaeo.2014.07.031>

(27) Hickman GC (1984) Swimming ability of talpid moles, with particular reference to the semi-aquatic Condylura cristata. Mammalia. 48(4):505–514.

(28) Sánchez-Villagra, M. R., Horovitz, I. & Motokawa, M. A comprehensive morphological analysis of talpid moles (Mammalia) phylogenetic relationships. *Cladistics* **22**, 59–88. <https://doi.org/10.1111/j.1096-0031.2006.00087.x> (2006).

(29) Hanawalt, F. 1922. Habits of the common mole: Scalopus aquaticus machrinus (Rafinesque). *The Ohio Journal of Science*, 22/6: 164-169.

(30) Sansalone, G., Kotsakis, T., Schwermann, A. H., Van den Hoek Ostende, L. W. & Piras, P. When moles became diggers: Tegulariscaptor gen. nov., from the early Oligocene of south Germany, and the evolution of talpid fossoriality. *J Syst Palaeontol* **16**, 645–657. <https://doi.org/10.1080/14772019.2017.1329235> (2017).

(31) Funmilayo, O. (1977) Distribution and abundance of moles (*Talpa europaea* L.) in relation to physical habitat and food supply. Oecologia. 30:277–283.

(32) Van den Hoek Ostende LW (2001) Insectivore faunas from the Lower Miocene of Anatolia. Part 8: Stratigraphy, palaeoecology, palaeobiogeography. Scripta Geol. 122: 101-122.

(33) Orr, P.J., Adler, L.B., Beardmore, S.R., Furrer, H., McNamara, M.E., Peñalver-Mollá E., Redelstorff, R., 2016. “Stick ‘n’ peel”: Explaining unusual patterns of disarticulation and loss of completeness in fossil vertebrates. Palaeogeography, Palaeoclimatology, Palaeoecology 457 (2016) 380–388. http://dx.doi.org/10.1016/j.palaeo.2016.05.024

(34) Hill, A., 1979. Disarticulation and scattering of mammal skeletons. Paleobiology, 5 (3), 1979, pp. 261-274.

(35) Reisdorf, A.G., Wuttke, M., 2012. Re-evaluating Moodie’s Opisthotonic-Posture Hypothesis in Fossil Vertebrates Part I: Reptiles—the taphonomy of the bipedal dinosaurs Compsognathus longipes and Juravenator starki from the Solnhofen Archipelago (Jurassic, Germany). Palaeobio Palaeoenv (2012) 92:119–168. DOI: 10.1007/s12549-011-0068-y
