# Supplementary material for: An unexpected Scalopini mole (Talpidae, Mammalia) from the Pliocene of Europe sheds light on the phylogeny of talpids
Source: Sci Rep. 2025 Jul 10;15:24928. doi: 10.1038/s41598-025-10396-1 (PMC12246231; doi:10.1038/s41598-025-10396-1)
Supplement: Supplementary file 2 — Supplementary Material 2 [file 41598_2025_10396_MOESM2_ESM.docx]

Supplementary Information 2 for “An unexpected Scalopini mole (Talpidae, Mammalia) from the Pliocene of Europe sheds light on the phylogeny of talpids”.

Adriana Linares-Martín, Marc Furió, Bruno Gómez de Soler, Jordi Agustí, Oriol Oms, Federica Grandi, Hugues-Alexandre Blain, Elena Moreno-Ribas, Pedro Piñero, Gerard Campeny

Tables of measurements and comparisons

|  | M1 | | M2 | | M3 | | P4 | | P3 | | Ref. |
| --- | --- | --- | --- | --- | --- | --- | --- | --- | --- | --- | --- |
|  | L | W | L | W | L | W | L | W | L | W |  |
| *Vulcanoscaptor ninoti* gen. et sp. nov. | 2.27 | 1.78 | 1.82 | 1.66 | 1.52 | 1.35 | 1.36 | 1.13 | 0.85 | 0.47 | This work |
| *Asthenoscapter meini* | 1.72 | 1.97 | 1.53 | 1.78 | 1.09 | 1.52 | 1.18 | 1.03 | - | - | (1) |
| *Desmanella gudrunae* | 1.86 | 1.41 | 1.39 | 1.74 | 0.94 | 1.34 | 1.27 | 1.05 | 0.45 | 0.39 | (2) |
| *Mygatalpa arvernensis* | 2.05 | 1.49 | 1.54 | 1.87 | 1.31 | 1.24 | 1.45 | 1.11 | 0.71 | 0.49 | (3) |
| *Mygalea magna* | 2.51 | 1.72 | 1.93 | 2.08 | 1.33 | 1.76 | 1.85 | 1.32 | 1.32 | 0.93 | (2) |
| *Archaeodesmana baetica* | 3.19 | 2.61 | 2.13 | 2.75 | 1.46 | 2.02 | 2.35 | 2.17 | 1.55 | 1.37 | (4) |
| *Scaptonyx edwardsi* | - | - | 1.54 | 2.05 | - | - | - | - | - | - | (1) |
| *Myoxomygale hutchisoni* | 2.02 | 1.42 | 1.52 | 1.76 | 1.03 | 1.48 | 1.44 | 1.12 | 0.63 | 0.47 | (5) |
| *Neurotrichus polonicus* | 2.37 | 2.70 | 1.90 | 2.45 | - | - | 1.70 | 1.22 | - | - | (6) |
| *Condylura kowalskii* | 2.02 | 1.98 | 2.00 | 1.73 | - | - | - | - | - | - | (7) |
| *Talpa minor* | 1.83 | 1.16 | - | - | 1.65 | 1.45 | - | - | - | - | (8) |
| *Skoczenia copernici* | 2.14 | 2.10 | - | - | - | - | 2.10 | 1.49 | - | - | (9) |
| *Mioscalops ripafodiator* | 2.53 | 1.20 | 1.92 | 2.34 | 1.81 | 1.08 | 1.60 | 1.24 | 0.89 | 0.63 | (10) |
| *Domninoides mimicus* | 3.60 | 3.40 | 3.30 | 3.10 | 2.20 | 2.50 | 2.40 | 2.30 | - | - | (11) |
| *Domninoides knoxjonesi* | 2.90 | 1.70 | 2.05 | 2.00 | 2.33 | 2.33 | - | - | - | - | (12) |
| *Hugueneya primitivus* | - | - | 1.95 | 2.20 | 1.25 | 1.80 | 2.13 | 1.58 | - | - | (13) |
| *Leptoscaptor bavaricum* | 2.27 | 1.78 | 1.80 | 2.09 | 1.19 | 1.65 | 1.46 | 1.14 | 0.87 | 0.59 | (14) |
| *Leptoscaptor robustior* | 2.36 | 1.72 | 1.92 | 2.02 | - | - | 1.54 | 1.27 | - | - | (14) |

**Table S1.** Measurements (mm) of the upper dentition of *Vulcanoscaptor ninoti* gen. et sp. nov. compared to the mean values from some other selected species of Talpidae. L: Length, W: Width. Data taken from (1) Hutchison (1974); (2) Van Den Hoek Ostende and Fejfar (2006); (3) Hugueney (1972); (4) Martín-Suárez *et al.* (2001); (5) Klietmann *et al.* (2015); (6) Skoczen (1980); (7) Skoczen (1993); (8) Rabeder (1972); (9) Rzebik-Kowalska (2014); (10) Hutchison (1968); (11) Freeman (1979); (12) Dalquest *et al.* (1996); (13) Van den Hoek Ostende (1989); (14) Ziegler (2003).

|  | m1 | | m2 | | m3 | | p4 | |  |  |  |
| --- | --- | --- | --- | --- | --- | --- | --- | --- | --- | --- | --- |
|  | L | W | L | W | L | W | L | W | m1-m3 | Depth of jaw | Ref. |
| *Vulcanoscaptor ninoti* gen. et sp. nov. | 2.04 | 1.33 | 1.92 | 1.32 | 1.54 | 1.07 | 0.88 | 0.59 | 5.54 | Below m1/m2: 1.44 | This work |
| *Asthenoscapter meini* | 1.44 | 1.01 | 1.67 | 1.03 | 1.39 | 0.87 | 0.91 | 0.60 | 4.43 | - | (1) |
| *Desmanella gudrunae* | 1.39 | 0.99 | 1.54 | 0.95 | 1.24 | 0.74 | 0.99 | 0.57 | - | - | (2) |
| *Mygatalpa arvernensis* | 1.63 | 1.13 | 1.65 | 1.31 | 1.36 | 1.06 | 1.05 | 0.63 | - | - | (3) |
| *Mystipterus pacificus* | 1.60 | 1.13 | 1.61 | 1.16 | 1.42 | 0.88 | - | - | 4.55 | 1.68 | (4) |
| *Theratiskos rutgeri* | 1.72 | 1.02 | 1.74 | 1.03 | 1.34 | 0.69 | 1.10 | 0.63 | - | - | (5) |
| *Gaillardia thomsoni* | 2.45 | 1.90 | 2.59 | 1.90 | 2.24 | 1.66 | - | - | 7.05 | - | (4) |
| *Mesoscalops scopelotemos* | 2.82 | 2.39 | 2.63 | 2.23 | - | - | - | - | - | - | (6) |
| *Mygalea magna* | 2.16 | 1.32 | 2.08 | 1.37 | 1.71 | 1.13 | 1.56 | 0.82 | - | - | (2) |
| *Mygalinia hungarica* | - | - | 2.12 | 1.30 | - | - | - | - | - | - | (7) |
| *Archaeodesmana baetica* | 2.63 | - | 2.44 | - | 1.92 | - | 2.18 | 1.48 | - | - | (8) |
| *Scaptonyx edwardsi* | 1.50 | 1.00 | 1.65 | 0.95 | 1.45 | 0.80 | - | - | - | 4.36 | (1) |
| *Mongoloscapter zhegalloi* | - | - | 2.00 | 1.50 | 1.65 | 1.30 | - | - |  | Below m2: 1.75 Below m3: 1.90 | (9) |
| *Talpa minor* | 1.95 | 1.20 | - | - | 1.50 | 0.75 | - | - | 5.40 | Below m2: 1.70 | (10) |
| *Talpa fossilis* | 2.40 | 1.46 | - | - | 2.00 | 0.96 | - | - | 6.63 | Below m2: 2.10 | (11) |
| *Skoczenia copernici* | 2.55 | 1.47 | 2.24 | 1.38 | - | - | 1.97 | 0.85 | - | Below m1: 2.08 | (11) |
| *Neurotrichus polonicus* | 1.79 | 1.18 | 1.93 | 1.14 | 1.76 | 0.98 | 1.22 | 0.74 | 5.57 | Below m2: 1.85 | (12) |
| *Myoxomygale hutchisoni* | 1.60 | 1.04 | 1.67 | 1.03 | 1.33 | 0.81 | 1.04 | 0.61 | 4.40 | - | (13) |
| *Condylura kowalskii* | 1.60 | 1.00 | 1.75 | 0.90 | 1.50 | 0.80 | - | - | - | - | (4) |
| *Mioscalops ripafodiator* | 1.77 | 1.43 | 2.14 | 1.46 | 1.83 | 1.16 | - | - | - | - | (4) |
| *Domninoides* sp. | 2.07 | 1.39 | 2.29 | 1.39 | - | - | - | - | - | - | (4) |
| *Domninoides knoxjonesi* | 2.18 | 1.33 | 1.70 | 1.30 | 1.75 | 1.05 | - | - | - | - | (14) |
| *Domninoides hessei* | 3.00 | 2.50 | 2.90 | 1.95 | 2.65 | 1.25 | - | - | - | - | (14) |
| *Domninoides mimicus* | 3.20 | 2.50 | 3.30 | 2.50 | 2.60 | 1.40 | 1.60 | 1.20 | - | - | (15) |
| *Scapanoscapter simplicidens* | 2.46 | 1.72 | 2.33 | 1.87 | 1.83 | - | - | - | 7.00 | - | (4) |
| *Scalopus (Hesperoscalops) mcgrewi* | 3.46 | 2.48 | 3.56 | 2.19 | 3.03 | 2.20 | 1.60 | 1.30 | - | - | (16) |
| *Scapanus (Xeroscapheus) procedirens* | 2.62 | 1.96 | 2.68 | 1.87 | 2.36 | 1.53 | - | - | 7.75 | Below m1: 3.50 | (4) |
| *Hugueneya primitivus* | 2.21 | 1.46 | 2.26 | 1.37 | 1.87 | 0.95 | 1.75 | 0.98 | - | - | (17) |
| *Achlyoscapter longirostiris*, sp.n. | 1.60 | 1.01 | 1.64 | 0.99 | - | - | 0.98 | 0.51 | 4.35 | Below m1: 1.57 | (4) |
| *Leptoscaptor bavaricum* | 1.68 | 1.10 | 1.90 | 1.20 | 1.64 | 1.00 | 1.04 | 0.63 | 5.21 | - | (18) |
| *Leptoscaptor robustior* | 1.85 | 1.11 | 2.04 | 1.22 | 1.74 | 1.02 | 1.11 | 0.61 | - | - | (18) |

**Table S2.** Measurements (mm) of the lower dentition of *Vulcanoscaptor ninoti* gen. et sp. nov. compared to the mean values from some other selected species of Talpidae. L: Length, W: Width. Data taken from (1) Hutchison (1974); (2) Van Den Hoek Ostende and Fejfar (2006); (3) Hugueney (1972); (4) Hutchison (1968); (5) Van den Hoek Ostende (1997); (6) Ostrander (1986); (7) Rzebik-Kowalska & Rekovets (2016); (8) Martín-Suárez *et al.* (2001); (9) Lopatin (2002); (10) Rabeder (1972); (11) Rzebik-Kowalska (2014); (12) Skoczen (1980); (13) Klietmann *et al.* (2015); (14) Dalquest *et al.* (1996); (15) Freeman (1979); (16) Voorhies (1977); (17) Van den Hoek Ostende (1989); (18) Ziegler (2003).

|  | Length | Distal width | Shaft thickness | Shaft widht (minimum diaphysal width) | Ref. |
| --- | --- | --- | --- | --- | --- |
| *Vulcanoscaptor ninoti* gen. et sp. nov. | 7.14* | 6.04 | 2.37 | 3.30 | This work |
| *Desmanella engesseri* | 7.13 | 3.07 | - | 0.92 | (1) |
| *Gaillardia thomsoni* | 18.90 | 11.4 | - | - | (2) |
| *Mesoscalops montanensis* | 9.30 | - | - | - | (3) |
| *Proscalops evelynae* | 8.40 | - | - | - | (3) |
| *Oligoscalops galbrethi* | 6.90 | - | - | - | (3) |
| *Scaptonyx edwardsi* | 7.05 | 6.90 | 3.36 | 1.07 | (4) |
| *Mygalea magna* | 12.50 | 6.50 | - | - | (5) |
| *Neurotrichus polonicus* | 8.48 | 4.31 | 1.58 | 1.70 | (6) |
| *Myxomygale hutchisoni* | 7.44 | 4.29 | 1.34 | 1.42 | (7) |
| *Condylura kowalskii* | 9.95 | 4.92 | 2.23 | 2.11 | (6) |
| *Mioscalops isodens* | 9.24 | 4.93 | - | 2.31 | (2) |
| *Mioscalops ripafodiator* | 9.49 | 4.51 | - | 2.95 | (2) |
| *Domninoides sp.* | 8.03* | 7.02 | - | 4.23 | (2) |
| *Parascalops fossilis* | 10.70 | 6.11 | - | 2.90 | (6) |
| *Parascalops breweri* | 12.30 | 7.20 | - | 3.70 | (6) |
| *Scapanoscapter simplicidens* | 12.50 | - | - | 4.20 | (2) |
| *Scapanus hagermanensis* | 12.40 | - | - | - | (8) |
| *Scapanulus oweni* | 12.30 | 5.7 | - | 2.50 | (6) |
| *Hugueneya* sp. | 11.50 | 8.33 | 3.4 | 3.65 | (9) |
| *Leptoscaptor bavaricum* | - | 4.65 | - | 1.83 | (10) |
| *Leptoscaptor robustior* | 10.30 | 5.45 | - | 2.67 | (10) |

**Table S3.** Measurements (mm) of the humerus of *Vulcanoscaptor ninoti* gen. et sp. nov compared to the mean values from some other selected species of Talpidae. This symbol (*) indicates that the humerus does not preserve its proximal end. Data taken from (1) Ziegler (1985); (2) Hutchison (1968); (3) Geisler (2004); (4) Skoczen (1980); (5) Van Den Hoek Ostende and Fejfar (2006); (6) Skoczen (1993); (7) Klietmann *et al.* (2015); (8) Hutchison (1987); (9) Lopatin (2003); (10) Ziegler (2003).

|  | | Proximo-distal Length | Proximal | Distal | | Ref. |
| --- | --- | --- | --- | --- | --- | --- |
|  | |  | Width | Width | Maximum thickness |  |
| *Vulcanoscaptor ninoti* gen. et sp. nov. | 7.56 | 2.57 | 2.77 | 1.56 | This work |  |
| *Mesoscalops scopelotemos* | 15.04 | 2.89 | 4.12 | - | (1) |  |
| *Neurotrichus polonicus* | 8.80 | 1.60 | 0.95 | 0.68 | (2) |  |
| *Condylura kowalskii* | - | 2.44 | 1.06 | 0.88 | (2) |  |
| *Mioscalops isodens* | 7.78 | - | 2.25 | - | (3) |  |
| *Mioscalops ripafodiator* | 7.86 | - | 2.23 | - | (3) |  |
| *Domninoides mimicus* | 10.6 | - | 5.20 | - | (4) |  |
| *Domninoides valentinensis* | 9.9 | - | 4.56 | - | (4) |  |
| *Proscapanus intercedens* | 7.93 | 1.74 | 2.74 | - | (5) |  |

**Table S4.** Measurements (mm) of the radius of *Vulcanoscaptor ninoti* gen. et sp. nov compared to the mean values from some other selected species of Talpidae. Data taken from (1) Ostrander (1986); (2) Skoczen (1980); (3) Hutchison (1968); (4) Freeman (1979); (5) Klietmann *et al.* (2015).

|  | Length | Shaft Length | Olecranon Length |
| --- | --- | --- | --- |
| *Vulcanoscaptor ninoti* gen. et sp. nov. | 11.46 | 6.4 | 4.11 |
| *Domninoides mimicus* | 21.11 | 11.11 | 8.80 |
| *Domninoides valentinensis* | 19.25 | 10.50 | 7.60 |

**Table S5.** Measurements (mm) of the ulna of *Vulcanoscaptor ninoti* gen. et sp. nov. compared to the mean values from *Domninoides* (see Freeman 1979).

|  | Length | Distal width | Shaft width | Shaft thickness |
| --- | --- | --- | --- | --- |
| *Vulcanoscaptor ninoti* gen. et sp. nov. | 7.14* | 5.99 | 3.30 | 2.37 |
| *Proscapanus primitivus* | 12.50 | 7.17 | 3.54 | 3.29 |
| *Proscapanus intercendens* | 10.30 | 6.00 | - | - |
| *Proscapanus sansaniensis* | 12.30 | 7.90 | - | - |
| *Proscapanus* cf. *primitivus* | 13.40 | 8.10 | - | - |

**Table S6.** Measurements (mm) of the humeri of *Vulcanoscaptor ninoti* gen. et sp. nov. compared to the mean values from selected species of *Proscapanus* (see Ziegler 1985).

|  | *Vulcanoscaptor ninoti* gen. et sp. nov. | | | *Proscapanus primitivus* | | |
| --- | --- | --- | --- | --- | --- | --- |
|  | Length | Trigonid W. | Talonid W. | Length | Trigonid W. | Talonid W. |
| p1 | 0.58 | 0.38 | - | 0.79 | 0.48 | - |
| p2 | 0.64 | 0.45 | - | 0.64 | 0.41 | - |
| p3 | 0.77 | 0.58 | - | 0.68 | 0.47 | - |
| m1 | 1.69 | 0.76 | 1.00 | 2.11 | 1.06 | 1.28 |
| m2 | 1.63 | 1.12 | 0.98 | 2.35 | 1.35 | 1.31 |
| m3 | 1.53 | 0.88 | 0.67 | 2.00 | 1.07 | 0.84 |
| m1-m3 | 5.20 | - | - | 6.63 | - | - |
| p1-m3 | 9.16 | - | - | 9.71 | - | - |
| i2-m3 | 10.03 | - | - | 11.81 | - | - |

**Table S7.** Measurements (mm) of the lower dentition of *Vulcanoscaptor ninoti* gen. et sp. nov compared to *Proscapanus primitivus* (see Hutchison 1974). W: Width.

|  | *Scapanulus oweni* from China (extant) | | | *Scapanulus oweni* from Shanyangzhai (Fossil) | | | *Vulcanoscaptor ninoti* gen. et sp. nov. | | | |
| --- | --- | --- | --- | --- | --- | --- | --- | --- | --- | --- |
|  | Length | Trigonid W | Talonid W | Length | Trigonid W | Talonid W | | Length | Trigonid W | Talonid W |
| m1 | 1.79 | 1.31 | 1.59 | 1.93 | 1.32 | 1.56 | | 1.69 | 0.76 | 1.00 |
| m2 | 2.07 | 1.66 | 1.55 | 2.17 | 1.45 | 1.44 | | 1.63 | 1.12 | 0.98 |
| m3 | 1.76 | 1.38 | 1.00 | 2.11 | 1.27 | 1.04 | | 1.53 | 0.88 | 0.67 |
| m1-m3 | 5.79 | - | - | 5.86 | - | - | | 6.63 | - | - |
| M1 | - | 1.93 | - | - | 1.60 | 1.60 | | - | 1.62 | 1.70 |
| M2 | - | 2.41 | - | - | 2.28 | 2.04 | | - | 1.93 | 1.52 |

**Table S8.** Measurements (mm) of the upper and lower dentition of *Vulcanoscaptor ninoti* gen. et sp. nov. compared to *Scapanulus* (see Li *et al.* 2016). W: Width.

|  | Length | Maximum W | Minimum W |
| --- | --- | --- | --- |
| *Scapanulus oweni* (extant) | 12.30 | 7.50 | 2.50 |
| *Scapanulus oweni* from Shanyangzhai | 13.00 | 7.90 | 2.75 |
| *Vulcanoscaptor ninoti* gen. et sp. nov. | 7.14* | 5.99 | 3.30 |

**Table S9.** Measurements (mm) of the humerus of *Vulcanoscaptor ninoti* gen. et sp. nov. compared to *Scapanulus* (see Li *et al.* 2016). W: Width.

References for Supplementary Information 2

Dalquest, W. W., Baskin, J., & Schultz, G. (1996). Fossil mammals from a late Miocene (Clarendonian) site in Beaver County, Oklahoma. Contributions in Mammalogy: A Memorial Volume Honoring Dr. J. Knox Jones, Jr. Museum of Texas Tech University, 107–137.

Freeman, P. W. (1979). Redescription and comparison of a highly fossorial mole, *Domninoides mimicus* (Insectivora, Talpidae), from the Clarendonian. American Museum Novitates, 2667, 1–16.

Geisler, J. H. (2004). Humeri of *Oligoscalops* (Proscalopidae, Mammalia) from the Oligocene of Mongolia. Bulletin of the American Museum of Natural History, 2004(285), 166–176. https://doi.org/10.1206/0003-090(2004)285<0166:c>2.0.co;2

Hugueney, M. (1972). Les talpidés (Mammalia, Insectivora) de Coderet-Bransat (Allier) et l’évolution de cette famille au cours de l’Oligocène et du Miocène inférieur d’Europe. Travaux et Documents Des Laboratoires de Géologie de Lyon, 50(1), 1–81.

Hutchison, J. H. (1968). Fossil Talpidae (lnsectivora, Mammalia) from the later Tertiary of Oregon. Bulletin of the Museum of Natural History, 11, 1–117.

Hutchison, J. H. (1974). Notes on type specimens of European Miocene Talpidae and a tentative classification of old world Tertiary Talpidae (Insectivora: Mammalia). Geobios, *7*(3), 211–256. https://doi.org/10.1016/S0016-6995(74)80009-4

Hutchison, J. H. (1987). Late Pliocene (Blancan) Scapanus (Scapanus)(Talpidae: Mammalia) from the Glenns Ferry Formation of Idaho. Museum of Paleontology, University of California. PaleoBios 12(45), 1-7.

Klietmann, J., Nagel, D., Rummel, M., & van den Hoek Ostende, L. W. (2015). A gap in digging: the Talpidae of Petersbuch 28 (Germany, Early Miocene). Palaontologische Zeitschrift, 89(3), 563–592. https://doi.org/10.1007/s12542-014-0228-2

Li, Y. xiang, Li, J., & Zhang, Y. xiang. (2016). Fossil Scapanulus oweni (Eulipotyphla, Mammalia) from the Shanyangzhai Cave, Middle Pleistocene, Qinhuangdao, China. Quaternary International, 392, 197–202. https://doi.org/10.1016/j.quaint.2015.08.001

Lopatin, A. V. (2002). An oligocene mole (Talpidae, Insectivora, Mammalia) from Mongolia. Paleontologicheskii Zhurnal, 36(5), 91–92.

Lopatin, A. V. (2003). Early Miocene Small Mammals from the North Aral Region (Kazakhstan) with Special Reference to their Biostratigraphic Significance. Paleontological Journal, 38(3), 217–323.

Martín-Suárez, E., Bendala, N., Freudenthal, M., & Freudenthal, M. (2001). *Archaeodesmana baetica*, sp. Nov. (mammalia, insectivora, talpidae) from the mio-pliocene transition of the granada basin, Southern Spain. Journal of Vertebrate Paleontology,https://doi.org/10.1671/0272-4634(2001)021[0547:ABSNMI]2.0.CO;2

Ostrander, G. E. (1986). Insectivora (Mammalia) from the Miocene (Hemingfordian) of western Nebraska. Transactions of the Nebraska Academy of Sciences, 14, 21–24.

Rabeder, G. (1972). Die Insectivoren und Chiropteren ( Mammalia ) aus dem Altpleistozän von Hundsheim (Niederösterreich) [Insectivora and Chiroptera (Mammalia) from the Pleistocene of Hundsheim (Niederösterreich)]. Annalen Des Naturhistorischen Museums in Wien, 76, 375–474.

Rzebik-Kowalska, B. (2014). Review of the Pliocene and Pleistocene Talpidae. Palaeontologia Electronica, 17(2), 1–26.

Rzebik-Kowalska, B., & Rekovets, L. I. (2016). New data on eulipotyphla (Insectivora, Mammalia) from the late Miocene to the Middle Pleistocene of Ukraine. Palaeontologia Electronica, 19(1), 1–31. https://doi.org/10.26879/573

Skoczen, S. (1980). Scaptonychini Van Valen, 1967, Urotrichini and Scalopini Dobson, 1883 (Insectivora, Mammalia) in the Pliocene and Pleistocene of Poland. Acta Zoologica Cracoviensia, 24(9), 411–448.

Skoczen, S. (1993). New records of *Parascalops*, *Neurotrichus* and *Condylura* (Talpinae, Insectivora) from the Pliocene of Poland. Acta Theriologica, 38(2), 125–137. https://doi.org/10.4098/AT.arch.93-11

Van den Hoek Ostende, L. W. (1989). The Talpidae (Insectivora, Mammalia) of Eggingen-Mittelhart (Baden-Wurttenberg, FRG) with special reference to the *Paratalpa-Desmanodon* lineage. Stuttgarter Beiträge zur Naturkunde. B, (152), 1–29.

Van den Hoek Ostende, L. W. (1997). Insectivore faunas from the Lower Miocene of Anatolia. Part 4: The genus Desmenodon (Talpidae) with the description of a new species from the Lower Miocene of Spain. Proceedings Koninklijke Akademie van Wetenschappen, 100, 27–65.

Van den Hoek Ostende, L. W, & Fejfar, O. (2006). Erinaceidae and Talpidae (Erinaceomorpha , Soricomor- pha , Mammalia) from the Lower Miocene of Merkur-Nord (Czech Republic , MN 3). *Beiträge Zur Paläontologie*, *30*, 175–203.

Voorhies, M. R. (1977). Fossil moles of late hemphillian age from ortheastern Nebraska. *Transactions of the Nebraska Academy of Sciences*, *4*, 129–138.

Ziegler, R. (1985). Talpiden (Mammalia, Insectivora) aus dem Orleanium and Astaracium Bayerns. *Mitteilungen Der Bayerische Staatssammlung Für Paläontologie Und Historische Geologie*, *25*, 131–175.

Ziegler, R. (2003). Moles (Talpidae) from the late Middle Miocene of South Germany. *Acta Palaeontologica Polonica*, *48*(4), 617–648.
