# Supplementary material for: An unexpected Scalopini mole (Talpidae, Mammalia) from the Pliocene of Europe sheds light on the phylogeny of talpids
Source: Sci Rep. 2025 Jul 10;15:24928. doi: 10.1038/s41598-025-10396-1 (PMC12246231; doi:10.1038/s41598-025-10396-1)
Supplement: Supplementary file 3 — Supplementary Material 3 [file 41598_2025_10396_MOESM3_ESM.docx]

Supplementary Information 4 for “An unexpected Scalopini mole (Talpidae, Mammalia) from the Pliocene of Europe sheds light on the phylogeny of talpids”.

Adriana Linares-Martín, Marc Furió, Bruno Gómez de Soler, Jordi Agustí, Oriol Oms, Federica Grandi, Hugues-Alexandre Blain, Elena Moreno-Ribas, Pedro Piñero, Gerard Campeny

List of characters used in cladistic analyses

Notes: Ordered characters (*)

## Teeth

1.* Number of upper incisors: three (0); two (1); one (2). [Modified from Sánchez-Villagra *et al.* (2006), character 2 and Hooker 2016 character 2; codings following Yates and Schmidly (1978); Kawada (2005); Sánchez-Villagra *et al.* (2006); Kawada *et al.* (2008)]

2. Size of anterior-most upper incisor: similar to the following (spatula-like) incisors (0); enlarged (1).

3.* Number of lower incisors: three (0); two (1); one (2). [Combination of Sánchez-Villagra *et al.* (2006), character 1 and 3; codings for *Scapanulus oweni* follow Li *et al.* (2016), compare supplement 3.]

4. Size of second lower incisor, I_2_: similar to other incisors (0); enlarged (1). [Following Ziegler (1971) the enlarged anterior-most lower incisor equates I_2_ in Talpidae. The anterior-most lower incisor in *Uropsilus* is treated as an I_2_. Whereas the dental formular of incisors and premolars is not clear in this taxon, the anterior-most lower incisor meets the characters of enlarged I_2_ in other talpids.]

5.* DP^1^ present (0); absent (1). [Sánchez-Villagra *et al.* (2006), character 4; coding reversed]

6.* DP_1_ present (0); absent (1). [Sánchez-Villagra *et al.* (2006), character 5; coding reversed]

7.* P^2^ present (0); absent (1). [Sánchez-Villagra *et al.* (2006), character 6; coding reversed]

8.* P_2_ present (0); absent (1). [Sánchez-Villagra *et al.* (2006), character 7; coding reversed]

9. P^3^ with two or more roots (0); single-rooted (1); absent (2). [Sánchez-Villagra *et al.* (2006), characters 8 and 9].

10. Relative size (height and width) of I^1^ and upper canine: canine larger (0); I^1^ larger (1). [Sánchez-Villagra *et al.* (2006)character 10; coding reversed].

11. P^4^, number of roots: three (0); two (1); one (2). [Sánchez-Villagra *et al.* (2006), character 11; coding reversed].

12. Upper canine, number of roots: one (0); two (1). [Modified from Sánchez-Villagra *et al.* (2006), character 13].

13. Upper canine: caniniform, without posterior crest (0); caniniform with posterior crest (1); premolariform (2). [Sánchez-Villagra *et al.* (2006), character 15].

14. M^2^ relative height of buccal cusps: subequal or paracone >metacone (0); metacone > paracone (1). [Sánchez-Villagra *et al.* (2006), character 14; coding reversed].

15. M^1^ metacone not expanded distolingually (0); expanded (1). [Sánchez-Villagra *et al.* (2006), character 16].

16. M^2^ paraconule present (0); absent (1). [Sánchez-Villagra *et al.* (2006), character 17; coding reversed].

17. M^2^ hypocone: absent (0); present distolingual with talon shelf (1); present mesially positioned with talon shelf (2) (Fig. 1C); mesially positioned without talon shelf (or if not distinguished, position on postprotocingulum) (3) (Fig. 1E). [Sánchez-Villagra *et al.* (2006), character 18, but homology corrected from metaconule to hypocone, following Butler (1988), and states 2 and 3 added.]

18. M^1^ mesostyle: absent (0); present single (1); present double, close to each other (2); present double, separated by deep valley (3). [Sánchez-Villagra *et al.* (2006), character 19]

19. M^2^ mesostyle: absent (0); present single (1); present double, close to each other (2); present double, separated by deep valley (3). [Sánchez-Villagra *et al.* (2006), character 20]

20. M^2^ postmetacrista and preparacrista subequal (0); postmetacrista longer (1). [Sánchez-Villagra *et al.* (2006), character 21; coding reversed. Both Sánchez-Villagra *et al.* (2006) and Schwermann and Thompson (2015) have used metacrista and paracrista respectively for these crests.]

21. Anterior accessory cuspid of M_2-3_ (Hutchison 1968, fig. 6): absent (0); present (1). [Modified from Sánchez-Villagra *et al.* (2006), character 22]

22. Lower molar precingulid: present (0); absent (1). [Sánchez-Villagra *et al.* (2006), character 23; coding reversed. N.B., soricids coded for presence contra Sánchez-Villagra *et al.* (2006): pers. obs. J. Hooker.]

23. Relative heights of M_1_ entoconid and metaconid: metaconid > entoconid (0); subequal (1). [Sánchez-Villagra *et al.* (2006), character 24]

24. M_1-2_ talonid notch: present (0); absent (1). [Sánchez-Villagra *et al.* (2006), character 25; coding reversed]

25. Position of attachment of M_2_ cristid obliqua to back of trigonid: lingual (0); central (1); buccal (2). [Modified from Sánchez-Villagra *et al.* (2006), character 26]

26. M_1-2_ talonid: with central hypoconulid on postcristid (0); nyctalodont (1); myotodont (2).

27. Upper premolar row: without gaps (0); with gaps (1). [Sánchez-Villagra *et al.* (2006), character 28; N.B. soricids coded for without]

28. Lower premolar row: without gaps (0); with gaps (1). [Sánchez-Villagra *et al.* (2006), character 42; N.B. soricids coded for without]

29. Contact between I^2^ and I^1^: present (0); absent (1). [Sánchez-Villagra *et al.* (2006), character 29; coding reversed]

30. Upper canine length: ≤ width (0); > width (1). [Sánchez-Villagra *et al.* (2006), character 31]

31. Height of P^4^: < upper canine (0); = upper canine (1); > upper canine (2). [Sánchez-Villagra *et al.* (2006), character 32; coding reversed]

32. P^4^ protocone large, mesiolingual of paracone (0); small to insignificant, lingual to distolingual of paracone (1). [Replaces Sánchez-Villagra *et al.* (2006), character 33, to which it adds data]

33. P^4^ parastyle: obvious (0); inconspicuous (1). [Sánchez-Villagra *et al.* (2006), character 34]

34. M^2^ length: similar to M^1^ length (0); < M^1^ length (1). [Sánchez-Villagra *et al.* (2006), character 35]

35. Length of M^3^: more than half M^1^ (0); less than half (1). [Sánchez-Villagra *et al.* (2006), character 36]

36. Crown area of M^3^: = or > P^4^ (0); < P^4^ (1). [Sánchez-Villagra *et al.* (2006), character 37]

37. DP^4^/_4_: functional (0); non-functional (1). Sánchez-Villagra *et al.* (2006), character 38]

38. The metacingulum of the M^2^: present (0); absent (1). [Sánchez-Villagra *et al.* (2006), character 38]

39. Posterior cingulum cusp of I_2_: absent (0); present (1). [Sánchez-Villagra *et al.* (2006), character 39; coding reversed]

40. P_4_ paraconid: present (0); absent (1). [Sánchez-Villagra *et al.* (2006), character 40]

41. P_4_ talonid: absent (0); unbasined with hypoconulid (1); basined with hypoconid and hypoconulid (2). [Modified from Sánchez-Villagra *et al.* (2006), character 41]

42. P_4_ metaconid: present (0); absent (1).

43. M^1^ preparacrista: present (0); absent (1).

44. Length of M_1_: subequal to P_4_ (0); longer but less than twice as long as P_4_ (1); more than twice as long as P_4_ (2). [Sánchez-Villagra *et al.* (2006), character 43]

45. Length of M_2_: < M_1_ (0); M_1_ and M_2_ subequal (1) (primitive); > M_1_ (2). [Sánchez-Villagra *et al.* (2006), character 44]

46. Length of M_3_: > or subequal to M_1_ (0); < M_1_ (1). [Sánchez-Villagra *et al.* (2006), character 45; coding changed]

47. M_2_ metastylid: absent (0); present (1). [Sánchez-Villagra *et al.* (2006), character 46]

48. M_2_ talonid width: < trigonid (0); sub-equal to trigonid (1). [Sánchez-Villagra *et al.* (2006), character 47; coding reversed]

49. M_1_ crown height, unworn to lightly worn: height of protoconid as percentage of tooth length: >90% (0); <90% (1). [Sánchez-Villagra *et al.* (2006), character 70; related crown height to dentary height. Here, both measurements are from the tooth, reducing the influence of other variables.]

## Cranium

50. Anterior nasal tip in lateral view: reaches level of incisors (0); level of canines (1); posterior to the posterior margin of canines (2). [Sánchez-Villagra *et al.* (2006), character 48]

51. Anterior extremity of incisive foramen: reaches level of I^3^ or more posterior (0); reaches level of I^2^ (1); anterior to the anterior margin of the I^2^ (2). [Sánchez-Villagra *et al.* (2006), character 49; polarity reversed]

52. Incisive foramina: small, anteroposterior length shorter than length of M^2^ (0); large, ≥ length of M^2-3^ (1). [Sánchez-Villagra *et al.* (2006), character 63]

53. Anterior extremity of anterior/major palatine foramina reaches: level of M^2^ (0); level of M^1^ (1); level of P^4^ (2). [Sánchez-Villagra *et al.* (2006), character 50; coding reversed]

54. Position of posterior border of infraorbital foramen relative to upper molar row: anterior to or at border of M^1^ and M^2^ (0); above M^2^ or more posterior (1). [Modified from Sánchez-Villagra *et al.* (2006), character 68]

55. Posterior margin of anterior root of zygomatic arch in ventral view extends: to M^3^ (0); to M^2^ (1). [Sánchez-Villagra *et al.* (2006), character 51; polarity reversed]

56. Zygomatic plate: dorsoventrally deep (0); shallow (1). [Motokawa (2004), character 6, fig. 2, compare Sánchez-Villagra *et al.* (2006), character 53; coding reversed]

57. In dorsal view, location of contact of zygomatic arch with braincase: medial to or at midpoint of anterior margin of braincase (0); at lateral portion of anterior margin of braincase (1); absent (2). [Modified from Sánchez-Villagra *et al.* (2006), character 55; coding reversed. Polarity based on ingroup commonality.]

58. Posterior margin of the infraorbital foramen: extends to M^1^ level or more anterior (0); extends to M^2^ level (1); extends to M^3^ level or more posterior (2). [Reworded and modified from Sánchez-Villagra *et al.* (2006), character 56; Motokawa (2004), character 12]

59. Zygomatic arch complete (0); broken (1). [Sánchez-Villagra *et al.* (2006), character 52]

60. Position of lacrymal foramen: posterior to infraorbital foramen (0); just dorsal or dorsal at the level of the middle portion of infraorbital canal (1); dorsal, just anterior to anterior border of infraorbital canal (2). [Sánchez-Villagra *et al.* (2006), character 62]

61. Foramen “I” in maxilla or premaxillary-maxillary suture: absent (0); present (1). [Sánchez-Villagra *et al.* (2006), character 64. Polarity based on ingroup commonality.]

62. Anterior projection of mastoid: well-developed, projecting laterally (0); weak to absent (1). [Sánchez-Villagra *et al.* (2006), character 58]

63. Anterior process of “mastoid process”: below root of zygomatic arch (0); in line with root (1); above root of zygomatic arch (2). [Sánchez-Villagra *et al.* (2006), character 67; coding reversed]

64. Position of posterior extremity of auditory bulla in ventral view: anterior to the anterior process of the “mastoid process” (0); in a similar position (1); posterior (2). [Sánchez-Villagra *et al.* (2006), character 57]

65. Maximal/minimal diameter of fenestra ovalis: <2.5 (0); >2.5 (1). [Sánchez-Villagra *et al.* (2006), character 71]

66. Stapes footplate: not bullate (0); bullate (1). [Sánchez-Villagra *et al.* (2006), character 72. Polarity based on ingroup commonality.]

67. Bony canal surrounding stapedial artery traversing the stapedial foramen: absent (0); canal partially or totally ossified (1). [Sánchez-Villagra *et al.* (2006), character 73. Polarity based on ingroup commonality.]

68.* Number of mental foramina: three or more (0); two (1); one (2). [Sánchez-Villagra *et al.* (2006), character 65; coding reversed]

69. Posterior mental foramen between P_3_ and P_4_ (0); between P_4_ and M_1_ (1); below M_1_ (2).

70. Posterior tip of the angular process of the dentary: anterior to condyle (0); approximately level with the condyle (1); posterior to the condyle (2). [Sánchez-Villagra *et al.* (2006), character 60]

71. Dentary angular process: plate-like (0); rod-like (1). [Modified from Sánchez-Villagra *et al.* (2006), character 66; coding changed for *Condylura*, where it is narrow but still plate-like, not rod-like.]

72. Position of mandibular condyle: between angular process and coronoid tip, but nearer angular process (0); at midpoint between upper sigmoid notch and coronoid tip or nearer coronoid tip (1). [Modified from Sánchez-Villagra *et al.* (2006), characters 59 and 61]

## Postcranial, axial

73. Axis and C3: not ankylosed (0); ankylosed (1). [Sánchez-Villagra *et al.* (2006), character 85]

74. Axis neural spine: cranio-caudally orientated keel (0); simple knob (1). [Sánchez-Villagra *et al.* (2006), character 86; coding reversed]

75. C6 transverse process posterior extension: does not reach C7-T1 border (0); reaches or surpasses this border (1). [Sánchez-Villagra *et al.* (2006), character 87]

76.* Number of caudal vertebrae: 20 or more (0); <20, >14 (1); 14 or fewer (2). [Sánchez-Villagra *et al.* (2006), character 88; coding reversed]

77. Sternum ventral surface: no distinct ridge (0); distinct ridge, but no keel (1); prominent keel (2). [Sánchez-Villagra *et al.* (2006), character 83. Polarity based on ingroup commonality.]

78.* Sternum, proportions of manubrium: length/width <1.5 (0); 1.5-3 (1); 3-4.5 (2); >4.5 (3). [Sánchez-Villagra *et al.* (2006), character 84. Polarity based on ingroup commonality.]

79. Dorsal surface of manubrium: smooth (0); contains a well-defined trough (1); features a ridge, sometimes perforated by a foramen (2). [Schwermann and Thompson (2015), character 158.ORD]

## Postcranial, appendicular, forelimb

80.* Clavicle: elongate, in some cases with strong processes directed medio-ventrally (0); semirectangular, stout (1); quadratic (length similar to width) (2). [Sánchez-Villagra *et al.* (2006), character 74. Polarity based on commonality in modern placentals.]

81. Clavicle, “foramen for vein”: absent (0); present (1). [Sánchez-Villagra *et al.* (2006), character 75. Polarity based on commonality in modern placentals.]

82.* Clavicle, articulations: with scapula (0); with scapula and humerus (1); with just humerus (2). [Sánchez-Villagra *et al.* (2006), character 76. Polarity based on commonality in modern placentals.]

83. Tetrahedral heterotopic bone wedged between ventromedial spine of the clavicle and anterior basilateral portion of the manubrium: absent (0); present (1). [Sánchez-Villagra *et al.* (2006), character 77. Polarity based on ingroup commonality.]

84. Scapula, suprascapular canal through base of acromion: absent (0); present (1). [Sánchez-Villagra *et al.* (2006), character 78. Polarity based on ingroup commonality.]

85. Scapula, infraspinous fossa: present (0); absent (1). [Sánchez-Villagra *et al.* (2006), character 79; coding reversed]

86. Scapula, marked teres fossa: absent (0); present (1). [Sánchez-Villagra *et al.* (2006), character 80]

87.* Scapula metacromion: absent (0); present < one third the length of the spine (1); present ≥ one third the length of the spine (2). [Sánchez-Villagra *et al.* (2006), character 81]

88. Scapula, coracoid process: conspicuous (0); inconspicuous (1). [Sánchez-Villagra *et al.* (2006), character 82; coding reversed]

89. Deltoid process of humerus: absent (0); present as flange distal to the greater tuberosity (1); present as elongate hook on lateral edge of greater tuberosity (2). [Sánchez-Villagra *et al.* (2006), character 89, states corrected by Schwermann and Thompson (2015)]

90. Position of humeral head: on posterior to posteromedial side of proximal end (0); lateral edge to centre of head in line with lateral edge of shaft (1); medial edge of head in line with lateral edge of shaft (2); entire head lateral to lateral edge of shaft (3). [Sánchez-Villagra *et al.* (2006), character 90, where state 3 was not scored. Some states have been recoded.]

91. Orientation of humeral head: long axis of head parallel or subparallel to shaft long axis (0); long axis of head at oblique angle to shaft long axis (1). [Sánchez-Villagra *et al.* (2006), character 91]

92.* Minimum width of humerus: approximately 1/9-1/10^th^ of maximum length of humerus (0); approximately 1/7^th^ (1); approximately 1/4-1/5^th^ (2); approximately 1/3^rd^ or less (3). [Sánchez-Villagra *et al.* (2006), character 92]

93. Distal end of pectoral crest of humerus: does not form pronounced and distinct process (0); forms pronounced and distinct process orientated proximo-medially (1); small but prominent process protruding at right angles to shaft (2). [Sánchez-Villagra *et al.* (2006), character 93; modified Schwermann and Thompson (2015)]

94. Proximity of pectoral crest to lesser tuberosity: clear gap with low proximal end of pectoral process (0); narrow gap or fused to form a bicipital tunnel (1). [Sánchez-Villagra *et al.* (2006), character 94]

95. Floor of bicipital groove: straight and parallel to long axis of humerus (0); displaced medially by pectoral crest near proximal end of humerus (1). [Sánchez-Villagra *et al.* (2006), character 95]

96. Open portion of proximal half of bicipital groove: visible in anterior view (0); visible in posterior view (1). [Modified from Sánchez-Villagra *et al.* (2006), character 96; where a state 2, “not visible”, was not scored]

97. Pit for m. flexor digitorum profundus: absent (0); present (1). [Sánchez-Villagra *et al.* (2006), character 97]

98. Medial edge of humeral trochlea: sharp, ventrally projecting ridge (0); straight or low ridge (1). [Sánchez-Villagra *et al.* (2006), character 98]

99. Lateral epicondyle: present as rounded protuberance (0); forms laterally extended flange (1); has proximally directed hook (2); has spine-like proximally pointed hook (3). [Sánchez-Villagra *et al.* (2006), character 99]

100. Brachial fossa: small pit (0); cavernous excavation underlying greater tuberosity (1). [Sánchez-Villagra *et al.* (2006), character 100]

101. Crest between greater tuberosity and distal end of pectoral ridge: present (0); absent (1). [Sánchez-Villagra *et al.* (2006), character 101]

102. Trough between head of humerus and greater tuberosity: very shallow to absent (0); deep groove (1). [Sánchez-Villagra *et al.* (2006), character 102]

103. Lesser tuberosity in posterior view: lower than proximal edge of head (0); level with proximal edge of head (1) higher than proximal edge of head (2). [Sánchez-Villagra *et al.* (2006), character 103; modified by Schwermann and Thompson (2015)]

104. Humeral head round (0); elliptical (1). [Sánchez-Villagra *et al.* (2006), character 104]

105. Scalopine ridge, running between the medial root of the humeral head and the distal margin of the lesser tuberosity: absent or weak (0); present as a distinct ridge or shelf (1). [Schwermann and Thompson (2015), character 171]

106. Medial epicondyle, proximally elongate flange or process: absent (0); present (1). [Sánchez-Villagra *et al.* (2006), character 105]

107. Greatest length of greater tuberosity and deltoid process: relatively short, c. <1/4 length of humerus (0); longer (1). [Sánchez-Villagra *et al.* (2006), character 106]

108. Pectoral crest: single straight process parallel to humerus long axis (0); forms single curved process (1); long axis of humerus and pectoral crest have perpendicular orientation (c.90 degrees) (2). [Sánchez-Villagra *et al.* (2006), character 107]

109. Clavicular facet: absent (0); in lateral view wedge-shaped (1); rectangular (2); sharp ending (3) [Sánchez-Villagra *et al.* (2006), character 26]

110. Lateral side of capitulum: not noticeably elongate (0); laterally elongate, so that capitulum has fusiform shape (1). [Sánchez-Villagra *et al.* (2006), character 109]

111. Teres tubercle of humerus: absent (0); a weak muscle scar (1); a distinct proximodistally short process (2); a distinct proximodistally elongate process (3). [Combined from Schwermann and Thompson (2015), characters 172, 173]

112. Ratio of the length of the olecranon process of the ulna to the length of the shaft: olecranon/shaft = <0.4 (0); >0.4 (1). [Schwermann and Thompson (2015), character 167]

113. Ulna, radial facet: flat to concave (0); convex, steep on lateral face (1). [Includes radial capitular process: Schwermann and Thompson (2015), character 170]

114. Ulna radial facet: confluent distally with humeral facet (0); separated distally by notch (1).

115. Ulna, proximal olecranon crest: absent (0); incipient, lateral process at level of anconal process (1) (Figs 4B, D, 5G); well-developed, but strongly oblique (2) (Fig. 5C, H); well-developed and nearly transverse (3) (Fig. 5D, E, I-J). [Modified from Schwermann and Thompson (2015), character 166]

116. Anterior tubercle of olecranon: absent (0) (Fig. 5A); present, strong, halfway along olecranon (1) (Fig. 5B); present, strong, at proximal end of olecranon (2) (Fig. 5C); present, weak towards proximomedial end of medial olecranon crest, where it meets the edge of the triceps area of insertion (3) (Fig. 5D); subsumed where the medial crest meets the triceps area of insertion at the medial extremity of the proximal olecranon crest (4) (Fig. 5E).

117. Ulna, anconal process: weak (0); strong (1).

118. Ulna coronoid process: weak (0); strong (1).

119. Abductor fossa and posterior crest of ulna: fossa narrow and very shallow, and crest a weak ridge (0); fossa deep, forming thin plate of bone and laterally curved forming a pronounced posterior crest (1). [Schwermann and Thompson (2015), character 168, where *Uropsilus* was coded ‘1’, despite having a very narrow fossa as noted by Hutchison, 1968: 15, fig. 4. Nevertheless, the depth in *Uropsilus* is greater than in soricids and appears to begin a trend towards ever broader and deeper fossae: Hutchison, 1968, fig. 13.]

120. Terminal process of the distal ulna (sensu Hutchison 1968), defined as an elongate posteriorly projecting process: absent (0); present (1). [Schwermann and Thompson (2015), character 169]

121.* Prepollex: absent (0); present as a knob (1); present, elongate, extending all along the scaphoid, but not reaching metacarpal I (2); present, extending to proximal portion of metacarpal I or beyond (3). [Sánchez-Villagra *et al.* (2006), character 110. Polarity based on commonality in modern placentals.]

122.* Scaphoid and lunar: not co-ossified (0); co-ossified, suture visible (1); co-ossified, suture not visible (2). [Sánchez-Villagra *et al.* (2006), character 111]

123. Cuneiform, ulno-palmar extension originating from distal portion: absent (0); present (1). [Sánchez-Villagra *et al.* (2006), character 112. Polarity based on commonality in modern placentals.]

124. Small sesamoid lateral to cuneiform: absent (0); present (1). [Sánchez-Villagra *et al.* (2006), character 113. Polarity based on ingroup commonality.]

125. Trapezium distinct distal arms: absent (0); present (1). [Sánchez-Villagra *et al.* (2006), character 114. Polarity based on ingroup commonality.]

126. Centrale: separate (0); absent/co-ossified (1). [Sánchez-Villagra *et al.* (2006), character 115; coding reversed]

127. Pisiform: simple (0); forms a plate larger in area than the cuneiform, extending palmar to the cuneiform, unciform and ulna (1). [Sánchez-Villagra *et al.* (2006), character 116. Polarity based on ingroup commonality.]

128. Proximal radial process of metacarpal I: absent (0); present (1). (Sánchez-Villagra and Menke 2005).

129. Length/width ratio of metacarpal IV: X6 (0); X4 (1); X2.5 (2); approximately as broad as long (3). [Partial overlap with Schwermann and Thompson (2015), character 174]

130. Ungual phalanges at least on fore foot: laterally compressed (0); relatively dorsoventrally compressed (1).

## Postcranial, appendicular, hindlimb

131. Fusion of acetabular area to vertebrae: absent (0); present (1). [Sánchez-Villagra *et al.* (2006), character 117. Polarity based on ingroup commonality.]

132.* Fusion of posterior horizontal branch of ischium to vertebrae: absent (0); transverse processes expanded but not fused to ischium (1); fused (2). [Sánchez-Villagra *et al.* (2006), character 118. Polarity based on commonality in modern placentals.]

133. Pubic approach: absent (0); pubes approach one another beneath acetabulum (1); pseudosymphysis formed (2). [Sánchez-Villagra *et al.* (2006), character 119; modified Schwermann and Thompson (2015). Polarity based on commonality in modern placentals.]

134. Pubic symphysis in the shape of a narrow bridge: absent (0); present (1). [Sánchez-Villagra *et al.* (2006), character 120. Polarity based on ingroup commonality.]

135. Femur, greater trochanter height: level with or below head (0); higher than head (1). [Sánchez-Villagra *et al.* (2006), character 121]

136. Third trochanter: absent (0); present, distal of lesser trochanter a small short flange (1); present, level with lesser trochanter, a small short flange (2); present, level with lesser trochanter, a short but broad and robust hooked flange (3). [Modified from Sánchez-Villagra *et al.* (2006), character 122 and Schwermann and Thompson (2015), character 176]

137. Tibia and fibula: separate or with syndesmosis (0); synostosed (1).

138. Tibial distal bridge: absent (0); present (1). [Sánchez-Villagra *et al.* (2006), character 123]

139. Tibial falciform process: absent (0); proximodistal blade (1); actual laterally projecting falciform process (2). [Sánchez-Villagra *et al.* (2006), character 124]

140.* Fibular lateral process: absent (0); simple lateral process (1); process with proximal head (2); process with proximal and distal heads (3). [Sánchez-Villagra *et al.* (2006), character 125]

141. Fibular posterior process: absent (0); present (1). [Sánchez-Villagra *et al.* (2006), character 126]

142. Astragalus, process on lateral side of body: absent (0); present (1). [Sánchez-Villagra *et al.* (2006), character 127]

143. Astragalar head width: narrower than body (0); as wide as body or wider (1). [Sánchez-Villagra *et al.* (2006), character 128]

144. Astragalar head, lateral side height relative to medial side: equal (0); lateral side higher (1). [Sánchez-Villagra *et al.* (2006), character 129]

145. Astragalar transverse ridge or groove proximal to trochlea: absent (0); ridge or groove (1). [Modified from Sánchez-Villagra *et al.* (2006), character 130. State 1, which is an autapomorphy of *Scalopus*, is combined with state 2.]

146. Astragalar proximoventral groove for the flexor digitorum fibularis tendon: shallow groove (0); deep groove or canal (1). [Sánchez-Villagra *et al.* (2006), character 131]

147.* Astragalar body proportions: mediolaterally wider (0); equidimensional (1); mediolaterally narrower (2). [Sánchez-Villagra *et al.* (2006), character 132]

148. Astragalar medial trochlear ridge orientation: proximodistal (0); proximally more lateral (1). [Sánchez-Villagra *et al.* (2006), character 133]

149. Distal end of astragalar lateral trochlear ridge ends on the distal end of the body (0); body is longer (1). [Sánchez-Villagra *et al.* (2006), character 134; but coded in reverse]

150. Astragalar medial plantar tuberosity: does not protrude medially beyond medial trochlear ridge (0); protrudes medially (1). [Sánchez-Villagra *et al.* (2006), character 135]

151. Astragalar neck angle with trochlea: large angle (0); small angle (1). [Sánchez-Villagra *et al.* (2006), character 136; coding reversed]

152. Astragalar proximoventral groove does not protrude proximally in dorsal view (0); protrudes proximally (1). [Sánchez-Villagra *et al.* (2006), character 137]

153.* Calcaneum, sustentacular facet dimensions: mediolaterally larger (0); equidimensional, round to square (1); proximodistally longer (2). [Sánchez-Villagra *et al.* (2006), character 138]

154. Calcaneum, peroneal process distal extent: proximal of or level with cuboid facet (0); protrudes distally (1). [Sánchez-Villagra *et al.* (2006), character 139]

155. Calcaneum, peroneal process lateral extent: protrudes laterally (0); does not protrude laterally (1). [Sánchez-Villagra *et al.* (2006), character 140; polarity reversed]

156. Peroneal process position: lateral to calcaneocuboid facet (0); dorsolateral to calcaneocuboid facet (1). [Sánchez-Villagra *et al.* (2006), character 141]

157.* Calcaneal cuboid facet, major axis: mediolaterally larger (0); equilateral axes (1); dorsoventrally larger (2); dorsoventrally much larger (3). [Sánchez-Villagra *et al.* (2006), character 142]

158. Ectal facet: without concave proximal extension (0); with concave proximal extension (1). [Sánchez-Villagra *et al.* (2006), character 143]

159. Peroneal process and sustentaculum proximodistal lengths: equal or peroneal process longer (0); peroneal process shorter (1). [Sánchez-Villagra *et al.* (2006), character 144 ; polarity reversed]

160. Ectocuneiform medial canal: absent (0); present (1). [Sánchez-Villagra *et al.* (2006), character 145]

161.* Navicular: tibial tuber weak to absent (0); short (1); long (2). [Reworded from Sánchez-Villagra *et al.* (2006), character 146]

162. Navicular ventral articular area: absent (0); ventral facet smaller than that on tuber (1); ventral and tuber facets subequal (2). [Sánchez-Villagra *et al.* (2006), character 147]

163. Navicular shape in dorsal view: mediolaterally wider (0); proximodistally longer (1). [Sánchez-Villagra *et al.* (2006), character 148]

164. Cuboid, medial proximal process: absent (0); present (1). [Sánchez-Villagra *et al.* (2006), character 149]

165. Cuboid ventrolateral tunnel: absent (0); present (1). [Sánchez-Villagra *et al.* (2006), character 150]

166. Cuboid proximal surface proximodistal location relative to that of the navicular: cuboid surface more distal (0); equal (1). [Sánchez-Villagra *et al.* (2006), character 151; polarity reversed]

167. Prehallux: absent or not in contact with entocuneiform and navicular (0); in contact (1). [Sánchez-Villagra *et al.* (2006), character 152]

168. Metatarsal I: with symmetrically opposite proximal and distal articulations (0); distal articulation twisted laterally (1).

169.* Length of metatarsal III relative to calcaneum: much longer, at least 1.31 times longer (0); between 1.16 and 1.01 times longer (1); shorter (2). [Sánchez-Villagra *et al.* (2006), character 153]

170. Metatarsal IV distal extent relative to M/T III: about equal or M/T IV shorter (0); M/T IV extends beyond M/T III (1). [Sánchez-Villagra *et al.* (2006), character 154]

171. Metatarsal V proximal lateral process: absent (0); terminal (1); subterminal (2).

172. Metatarsal V cuboid facet: present, large (0); present, small (1); absent (2).

173.* Number of mammae: three pairs (0); four pairs (1); five pairs (2). [Sánchez-Villagra *et al.* (2006), character 155]

174. Position of nostrils: anterior (0); lateral (1); superior (2). [Sánchez-Villagra *et al.* (2006), character 156]

175. Tail: scaly (0); not scaly (1). [Sánchez-Villagra *et al.* (2006), character 157]

Character-taxon matrix

Notes: Polymorphism: a = (01), b = (12), c = (23), d = (123), e = (02), f = (03).

Missing data = ?

## MATRIX

*Erinaceus*

011011a0a1 0a0001?001 0100??1111 201111?111 0?010101?0 2010111000 01??000112 2000020000 0000000010 0000000000 0000000000 000?????00 02?1010??? 0000011000 0000101001 102a102010 000001??00 ??211

*Solenodon cubanus*

010111a101 01?0?1100? 0100??0001 100111?101 0??11000?0 20?00?1011 0011?000?2 0?10000000 0000001010 0000000000 0000000000 100?????00 0000010??? 011101?000 001?001000 10?011?110 ??0?000?10 ??010

*Crocidura*

0120111121 00210a1111 1000210001 20011111?1 0001010000 102011201 011?2000212 10101200?0 0000002010 0010000000 1000000000 1?00220000 1000010000 0000011110 0000011001 0020001101 0000000100 10011

*Sorex*

0120110121 0221011111 010021000 020011111?1 2001010011 10b0112010 01?2000222 1000110000 0000002010 0210000000 1001000100 1000221000 0200010100 0000011110 0000011000 0000000111 0000000100 10011

*Blarina*

0120110121 0221011111 0100210000 20011111?1 2102010010 1020112010 01?2000221 10001210?00 000002010 0210000000 1001000100 3?10220000 0???010010 0000011110 0000111000 0000000111 0000000100 ???11

*Eotalpa*

????0?0?0? 00?000fdd1 1000120??0 ?0000001?0 100111011? ???0???0?? ????????20 0????????? ?????????? ?????????? ?????????? ??1111001? ???????111 ??????00?? ?100001010 010100100? ???0?1?11? 20???

*Uropsilus*

11b0000021 0001002111 0110120010 21011a01a1 1111b10110 b000111001 110a000120 1001101000 0100001011 1000100000 00100001?0 2000000010 0200000000 000011101a 0100000010 10000a2000 0111010100 10?10

*Tegulariscaptor minor*

??0??0?00? 0??1003111 00000200?? ?001101??0 111121011? ?????????? ???????12? ?????????? ????????12 1220101021 0121110111 2????????? ?????????? ?????????? ?????????? ?????????? ?????????? ?????

*Urotrichus*

011100??01 00010031b0 1111120010 2100000111 1111110110 2011100101 0112100122 0000a22000 a200011112 1121111021 0121010131 3000331010 2011000021 0b00011011 1101010010 1120112?11 1101010011 11011

*Dymecodon*

011000??01 0001003110 0111020010 2100001111 11112101102 011000201 0102000a22 0011a120?0 0200010111 11e1101??1 01b1??0131 ?????????? ?11?000011 01b0?110?? ?101002010 1021112001 211101??01 ??011

*Mygalea_jaegeri*

??00?0000? 0???0??cd0 01?12200?? ?10000?100 111111011? ?????????? ???????121 b0????2000 02?1??b1?3 0201101020 11b10101d0 3000331010 ?????????? ??????10?? ?????????? ?????????? ?????????? ?????

*Desmana*

0101000011 0101003331 0000020010 2100001101 0111110110 2110111101 0000101022 0100101000 0201012111 1101101010 0121010110 3000330010 0001000110 0101131022 1101102011 1121013010 1101101001 01100

*Galemys*

0101000001 0100003330 1100020010 1100001101 1111110110 211111110 11000001021 0001101000 a20a012111 1101101010 01210101?1 3000330010 2000000110 0101131023 1101102111 1121013010 110111?001 01120

*Scaptonyx*

0010000000 2100003211 1101021001 0100001100 1111201100 1011010201 1112000222 0001112101 0200011123 02211110c1 11210111d1 3000130010 2011000121 0100121012 1111000010 1121012011 2111111011 21?11

*Mongoloscapter zhegalloi*

?????????? ?????????? ?0??0??0?? ?????????? ??????11?? ?????????? ?????????? ?????????? ?????????? ?????????? ?????????? ?????????? ?????????? ?????????? ?????????? ?????????? ?????????? ?????

*Neurotrichus*

010011??01 1111013121 0000121001 2100010101 1110110110 1011010202 1112100122 0011112101 0200011123 1221101021 1121010111 3010330010 2011000021 0000121012 111110a010 112a012001 1101111011 20111

*Condylura cristata*

0000000001 0000003111 0001021100 2100001000 1001111112 0012010202 11000?1112 1110112201 0200011123 1201111031 0121010211 2011331011 3011000121 0110121013 1111100010 1020112010 1101011111 22101

*Geotrypus montisasini*

??10?1010? 0??00??bb1 01001210?? ?1?000?101 111111011? ?????????? ????????20 0?????2??2 02?0??112c 0301111031 01210112?1 31103?111? ?0??????31 ?????????? ?????????? ?????????? ?????????? ?????

*Geotrypus antiquus*

0000?00000 0100003221 1000121101 010000?100 1111210111 ????0??10? ???????122 11???????1 02?0??1123 02211110c1 11210112d1 3?002?11?0 301?????31 ????11???? ?????????? ?????????? ?????????? ?????

*Euroscaptor*

0000000000 0110003221 1100120001 010000110a 1111110a0a 0011010202 011b?1?111 0000022212 1200110122 0201111031 0011011211 3111331111 3010101031 1010011?23 1011000111 1120012011 1101111111 20?01

*Talpa*

0000000000 0111003121 1100120001 0100001100 1111210111 0011010202 1112001112 0000022212 a200110123 0201111031 0011011211 3111331111 3110101031 1010011023 1101000110 1010002011 1201111010 20a01

*Scaptochirus*

0000001100 0111013221 1100121001 0110001100 1111110101 001011110a 1120001120 0000022212 1200110123 0301111031 00110112d1 3111341111 3110101031 1210011023 1011000110 1021002001 2111111021 20?01

*Skoczenia copernici*

??0????0?? ???????22? ?1???????? ?????????? ????11?1?? ?????????? ???????12? ?????????? ?????????? ?????????? ?????????? ?????????? ?????????? ?????????? ?????????? ?????????? ?????????? ?????

*Parascaptor leucura*

0000001000 0110003221 1100021001 0100001101 1111110000 001b010201 11e1001211 0110022112 1200110123 0201111031 00110112d1 3111331111 3110101031 1020011023 1011000010 1010001011 2111111120 20?01

*Mogera*

0010000000 0111003221 1100120001 0100001101 1111110100 0011000102 1110011111 0010022212 1200110123 0201111031 0011011211 3111341111 3110101031 1220011023 1101100110 1110012001 2101111011 20101

*Proscapanus sansaniensis*

0101?00001 0100003cd? 10001200?? ?10001?101 111111011? ?????????? ???????11? ??????2??2 02?1??0122 03011110c1 0?211112d1 31??3?1111 ?01?00???1 ?????????? ?????????? ?????????? ?????????? ?????

*Scalopus aquaticus*

0111110011 2001110331 0110121110 2110001101 1012110100 2011001202 0102101012 0000022312 1201010123 0301111131 0021111221 3111341111 3110000031 1220011023 1111101011 1101112011 1101110021 21021

*Scapanus*

0101000011 2000113331 1110021010 2100001101 1112110100 1011001102 0102101112 0000022312 0201010121 0301111131 0021111221 3111331111 3010000031 1210011023 1111001010 1020002001 1101111?21 10121

*Scapanoscapter simplicidens*

??0????0?? ??????????  ?0??0??0?? ?????????? ?????11??? ?????????? ??????10?? ?????????? ?????????? ?????????? ?????????? ?????????? ?????????? ?????????? ?????????? ?????????? ?????????? ?????

*Domnionides mimicus*

a?b?01001? a0??013dd0 11?102?0?0 211000?101 111111111? ?0110??1?? ???????10? 0?????2222 02?1??01?? ??0???1021 0????1???1 311?3?1111 ?????????? ????01???? ?111?01010 1?200??01? ?????????? ?????

*Alpiscaptulus medogensis*

0001110001 ??011??33? ?1????1011 2?111????? ???110?1?0 201101010? ?0000???02 00???????? ?????????? ?????????? ?????????? ?????????? ?????????? ?????????? ?????????? ?????????? ?????

*Scapanulus oweni*

1111110001 0101013331 1110021010 2100001101 1111211100 2011010102 110100a202 0000022222 0211010122 0201101021 00211112d1 3?11331111 2010001031 0210121023 1111100010 1021112001 2101111121 10?11

*Hugueneya*

???????0?? ?????0??3? ?0??2??0?? ??0??????1 ?1???00??? ?????????? ?????????? ?????????? ?????????? ?30???10?? ??2??????1 3????????? ?????????? ?????????? ?????????? ?????????? ?????????? ?????

*Parascalops breweri*

0101000011 0000003331 1a10021010 2100001001 1012111110 1011010102 11120?0202 0000022222 0211010123 0301111031 0021111221 3111341111 3010001031 0110121023 1111100011 1111012011 1101111?20 ??111

*Mioscalops isodens*

??0??100?? ?????03?b1 ????02???? ???????1?0 1??12011?? ?????????? ???????1b? ?????????b ???1?10113 02011010?1 10211112?1 3?1?3?1011 ?????????? ?????????? ?????????? ?????????? ?????????? ?????

*Mioscalops ripafodiator*

??01?1?00? 0??0003221 001?02?1?? ?1010??101 111120110? ?????????? ???????11? ??????2??1 02?1?10113 0201101021 10211?12?1 3????????? ?0???????? ??????10?? ??1?101000 10???????? ?????????? ?????

*Leptoscaptor bavaricum*

211?01000? 0a?0003220 00??0211?? ?10100?1?1 1?110001?? ?????????? ???????b0? ?????????? ?2??????1? 0b01101??1 1??11?12?? 3?????11?? ?????????? ?????????? ?????????? ?????????? ?????????? ?????

*Yanshuella primaeva*

0100110001 000?0?3120 000?12?1?0 ?10100?001 101111010? ?????????? ???????12? ?????????? ????????23 0201101011 10211112?1 3?1???11?1 ?????????? ?????????? ?????????? ?????????? ?????????? ?????

*Yunoscaptor scalprum*

??21?0?0?? ????01??10 000?02???? ?11100?001 11012111?? ?????????? ???????11? ?????????? ?????????3 02?1101031 ?0211101?1 2????????? ?????????? ?????????? ?????????? ?????????? ?????????? ?????

*Vulcanoscaptor ninoti* gen. et sp. nov.

??111100?? 1??0103330 00110200?? ?00110?101 111101010? ?????????? ???????122 00???????? ?????????? ?3????11?? ?????????1 ?1113?1111 301000003? ?????????? ?????????? ?????????? ?????????? ?????

*Myxomygale*

0?0???00?? 0???00?110 ?11????0?? ?1000????? ?0?011?0?? ?????????? ???????1?? ?????????? ????????12 11????102? ??21000?20 ??????1?0? ?????????? ?????????? ?????????? ?????????? ?????????? ?????

References for Supplementary Information 4
