# Supplementary material for: An unexpected Scalopini mole (Talpidae, Mammalia) from the Pliocene of Europe sheds light on the phylogeny of talpids
Source: Sci Rep. 2025 Jul 10;15:24928. doi: 10.1038/s41598-025-10396-1 (PMC12246231; doi:10.1038/s41598-025-10396-1)
Supplement: Supplementary file 4 — Supplementary Material 4 [file 41598_2025_10396_MOESM4_ESM.docx]

Supplementary Information 1 for “An unexpected Scalopini mole (Talpidae, Mammalia) from the Pliocene of Europe sheds light on the phylogeny of talpids”.

Adriana Linares-Martín, Marc Furió, Bruno Gómez de Soler, Jordi Agustí, Oriol Oms, Federica Grandi, Hugues-Alexandre Blain, Elena Moreno-Ribas, Pedro Piñero, Gerard Campeny

Differential diagnosis

*Vulcanoscaptor ninoti* gen. et sp. nov. has been placed within the family Talpidae according to the following traits described by Hutchison^1^ and Hillson^2^: 1) the presence of a characteristic anterior extremities associated to fossorial ability; 2) the morphology of the lower mandible; 3) long and narrow skull and 4) the invariable presence of the three lower and upper molars along with the upper canine. These characters differentiate *Vulcanoscaptor ninoti* gen. et sp. nov. from all the small mammals belonging to any other family of similar size. The new taxon can be differentiated from other subfamilies and tribes using a unique combination of characters.

*Vulcanoscaptor* gen. nov. mainly differs from all Proscalopidae by not having a flexure of the maxillary between P3 and P4 (see Hutchison^1^), and by an overall smaller size (Tables S2–S4 in Supplementary Information 2). Other than that, proscalopids frequently show some characters not found in *Vulcanoscaptor ninoti* gen. et sp. nov., like: 1) three-rooted P4; 2) a distally pointed capitulum and a large fossa for the flexor digitorum muscle and 3) a flat, medially expanded ridge medial epicondyle in the humerus.

*Vulcanoscaptor* gen. nov. differs from *Eotalpa* by its P4 with a smaller parastyle and less prominent mesiolingual protocone lobe; by having an M1 and M2 less labiolingually elongated and by having its a fused tibiofibula^3,4,5^. *Vulcanoscaptor* differs from the *incertae sedis* form *Tegulariscaptor minor* by its more robust humerus; by the reduction of one lower incisor; by having an m1 with the oblique cristid of reaching the lingual margin; and by having a parastyle of the M2 less developed^6,7^. *Vulcanoscaptor* differs from the *incertae sedis* genus *Mongolopala* by having more pronounced hypoflexids, by having oblique cristids in m1 and m2 ending lingually, and by having more rounded and smaller M3 (see Ziegler^8^).

*Vulcanoscaptor* gen. nov. differs from *Desmanodon* by having an anterolingual extended postparacrista in M1 and M2; having oblique cristid in m1 and m2 ending lingually; a short and robust humerus; and having a straight medial edge of the humeral trochlea^9,10^.

*Vulcanoscaptor* gen. nov. differs from all the members of the subfamily Uropsilinae (*Uropsilus*, *Desmanella*, *Asthenoscapter*; *Mygatalpa*, *Mystipterus*; *Theratiskos*) by the absence of slender and narrow humeri, three-rooted P4s, undivided mesostyles in M1 and M2, and rounded upper molars in occlusal view with less pronounced lingual cusps. *Vulcanoscaptor* also differs from uropsilines by presenting a fossa for M. flexor digitorium profundus ligament in the humerus, a proximal olecranon crest in the ulna, and a capitular process in the radius^1^. In the uropsilines it is also frequent to find a sharp ventrally projecting ridge medial edge of the humeral trochlea. *Vulcanoscaptor* differs from the species included in the subfamily Gaillardinae, by the same set of traits as in Uropsilinae, in addition to their robust and wider ascending and horizontal rami of the mandible.

*Vulcanoscaptor* gen. nov. differs from desmanines (*Archaeodesmana*, *Desmana*, *Galemys*, *Mygalea*, *Mygalinia*) by having a less bulbous and smaller dentition, by having only two lower incisors, by having upper molars with poorly developed lingual cusps, by having a more robust humerus; by the presence of coronoid process in the ulna, and by having the capitular process in the radius rather well developed, other than an overall smaller size.

*Vulcanoscaptor* gen. nov. differs from the Urotrichini genera (*Urotrichus,* *Dymecodon*, *Tenuibrachiatum*, *Myxomygale*) by: having a slightly divided mesostyle in M1; the straight medial edge of the humeral trochlea; having a more robust humerus; the presence of well-developed coronoid processes, and; a dorsal extremity of the tibiofibulae not expanded into a hatchet-shaped structure.

*Vulcanoscaptor* gen. nov. differs from *Paratalpa* by: having a small and not laterally extended olecranon fossa; a short and robust humerus; and having a straight medial edge of the humeral trochlea^10^.

*Vulcanoscaptor* gen. nov. differs from the species of Scaptonychini (*Scaptonyx*) by: having a divided mesostyle in M2; a double-rooted P4 with parastyle; a posterior lower incisor longer than the anterior one; the absence of metastylid in m2; the straight medial edge of the humeral trochlea, and; the well-developed anconal and coronoid processes in the ulna.

*Vulcanoscaptor* gen. nov. differs from the known Neurotrichini genera (i.e., *Neurotrichus*, *Quyania* sensu Rzebik-Kowalska^11^) by preserving p1 and p2 (see Sansalone^7^), and by having a more robust and wider humerus. Moreover, *Vulcanoscaptor* differs from *Neurotrichus polonicus* in the area of the trochlea which is reduced by a great development of the lateral epicondyle in the latter species, also displaying a dorsal extremity expanded into a hatchet-shaped structure in the tibiofibulae.

*Vulcanoscaptor* gen. nov. differs from the Condylurini (*Condylura*) by: not having lower premolars separated by diastemata; doubled mesostyle in M1 and M2; the two-rooted P4; the absence of metastylid in m2; the straight medial edge of the humeral trochlea, and; the well-developed anconal process in the ulna.

*Vulcanoscaptor* gen. nov. differs from the Talpini (*Talpa, Geotrypus, Euroscaptor, Mogera, Parascaptor, Scaptochirus, Skoczenia*) by: a doubled mesostyle in M2; the double-rooted P4; the straight medial edge of the humeral trochlea; the absence of third lower incisors (with the exception of *Moguera* and *Geotrypus montisasini*), and; having hypertrophied lower anterior teeth. The studies of Sánchez-Villagra *et al.*^12^ and Schwermann *et al.*^13^ suggest the presence of the hypocone in M2 for the species of the talpini tribe. In the case of *Vulcanoscaptor* gen. nov., this trait is visible in M1 an M2.

The widening of the trochlear zone of the humerus, the mandibular processes, and the presence and strong development of the anconal and coronoid processes of the ulna place *Vulcanoscaptor ninoti* gen. et sp. nov. into the Scalopini. Within the tribe Scalopini there is no trait alone to distinguish the specimen herein described from all the rest of scalopine moles. However, the combination of characters is unique within the tribe. The following dental and postcranial traits must be considered to identify the species:

*Vulcanoscaptor* gen. nov. differs from *Mioscalops* by: having only two lower incisors; the postmetacrista is shorter than the preparacrista in M2; an m1 larger than m2; having a slightly wider humerus, and; the straight medial edge of the humeral trochlea.

*Vulcanoscaptor* gen. nov. differs from *Domninoides* by: having a metacone expanded distolingually in M1; the presence of paraconule in M2; the absence of an anterior accessory cuspid in m3; the presence of precingulids in lower molars, and; the absence of metastylid in m2.

*Vulcanoscaptor* gen. nov. differs from *Scapanulus* by: a double-rooted P4; a metacone more distolingually expanded in M1; a postmetacrista shorter than the preparacrista in M2; the absence of an anterior accessory cuspid of m2-m3; the presence of lower molar precingulid; a talonid notch absent in m1-m2; a m1 larger than m2, and; a straight medial edge of the humeral trochlea.

*Vulcanoscaptor* gen. nov. differs from *Parascalops* by: having only two lower incisors; a double-rooted P4; having a metacone of M1 rather expanded distolingually; a postmetacrista shorter than the preparacrista in M2; the absence of an anterior accessory cuspid in m2-m3; m2-m3 without talonid notches; the absence of gaps in the upper premolar row; the absence of metacingulum in M2; having a p4 without metaconid, and; having a straight medial edge of the humeral trochlea.

*Vulcanoscaptor* gen. nov. differs from *Scapanoscapter* by: only having two lower incisors; displaying an overall smaller size in both upper and lower molars.

*Vulcanoscaptor* gen. nov. differs from *Scalopus* by: having a double-rooted P4; a metacone lower than the paracone in M2; the presence of paraconule in M2; an M2 with a postmetacrista shorter than the preparacrista; the presence of lower molar precingulids; the absence of talonid notches in m1-m2; the absence of gaps in the upper and lower premolar toothrows; the presence of an advisable parastyle in P4; the absence of metaconid in p4, and; the absence of co-ossified scaphoid and lunar bones.

*Vulcanoscaptor* gen. nov. differs from *Scapanus* by: the presence of only two lower incisors; having an M2 with a postmetacrista shorter than the preparacrista; the absence of an anterior accessory cuspid in m2-m3; the presence of precingulid in the lower molars; the absence of talonid notches in m1-m2; the absence of gaps in the upper premolar rows, and; a less hypsodont lower molars.

*Vulcanoscaptor* gen. nov. differs from *Proscapanus* by: the presence of only two lower incisors; a rather expanded distolingually metacone in M1; the absence of an anterior accessory cuspid in m2-m3; having a metaconid lower than entoconid in m1; the absence of the talonid notch in m1-m2; and having a straight medial edge of the humeral trochlea.

*Vulcanoscaptor* gen. nov. differs from *Alpiscaptulus*: the presence of only two lower incisors; a metacone lower than paracone in M2; the presence of lower molar precingulids; the absence of gaps in the lower premolar row, and; the posterior tip of the angular process of the dentary not extending as far posteriorly as the condyle.

*Vulcanoscaptor* gen. nov. differs from *Hugueneya* by: having a p1 to p3 series gradually increasing in size; not showing well-developed entocristids in lower molars; the absence of a large entostylid in m2; the absence of broad posterior cingulids in m3; m1 without remarkable parastylid and entostylid; the absence of a posterior cingulum in M2, and; having a straight medial edge of the humeral trochlea.

*Vulcanoscaptor* gen. nov. differs from *Leptoscaptor* by: the double-rooted P4; a rather expanded distolingually metacone of the M1, and; the absence of gaps in the upper and lower premolar rows.

*Vulcanoscaptor* gen. nov. differs from *Yanshuella* by: the presence of only two lower incisors; having less blunt lower premolars with no marked labial cingulids; having a double-rooted P4; having a slightly divided mesostyle in M1; having oblique cristid of m2 reaching the lingual margin, not the protocristid; a metaconid lower than the entoconid in m1; having p2 and p3 without well-marked cingulids; the absence of gaps in the lower premolar row, and; having a straight medial edge of the humeral trochlea.

*Vulcanoscaptor* gen. nov. differs from *Yunoscaptor* by: the presence of one more lower incisor; a slightly divided mesostyle in M2; the presence of a parastyle in P4; the absence of a preparacrista in M1; the absence of a metastylid in m2, and; having a straight medial edge of the humeral trochlea.

References for Supplementary Information 1

1. Hutchison JH (1968). Fossil Talpidae (lnsectivora, Mammalia) from the later Tertiary of Oregon. Bull Mus Nat Hist Univ Oreg. (11):1–117.
2. Hillson, S. (2005). Teeth. Cambridge university press.
3. Sige, B., Crochet, JY., & Insole, A. (1977). Les plus vieilles taupes. *Geobios*, *10*, 141-157.
4. Hooker, JJ (2016). Skeletal adaptations and phylogeny of the oldest mole Eotalpa (Talpidae, Lipotyphla, Mammalia) from the UK Eocene: The beginning of fossoriality in moles. *Palaeontology* 59, 195–216.
5. Smith, R (2007). Présence du genre Eotalpa (Mammalia, Talpidae) dans l’Oligocène inférieur de Belgique (Formation de Borgloon, MP 21). Bulletin de l’Institut royal des Sciences naturelles de Belgique, Sciences de la Terre 77, 159–165.
6. Ziegler R (2012). Moles (Talpidae, Mammalia) from early Oligocene karstic fissure fillings in South Germany. Geobios 45:501–513.
7. Sansalone G, Kotsakis T, Schwermann AH, Van den Hoek Ostende LW, Piras P (2017). When moles became diggers: Tegulariscaptor gen. nov., from the early Oligocene of south Germany, and the evolution of talpid fossoriality. J Syst Palaeontol. 19:1-13. <http://doi.org/10.1080/14772019.2017.1329235>
8. Ziegler, R., Dahlmann, T., & Storch, G. (2007). Oligocene–Miocene vertebrates from the Valley of Lakes (Central Mongolia): morphology, phylogenetic and stratigraphic implications. 4. Marsupialia, Erinaceomorpha and Soricomorpha (Mammalia). Annalen des Naturhistorischen Museums in Wien A, 108, 53-164.
9. Prieto, J. (2010). The Middle Miocene mole Desmanodon crocheti sp. nov.(Talpidae, Mammalia): the last representative of the genus in the North Alpine foreland basin. Paläontologische Zeitschrift, 84, 217-225.
10. Van den Hoek Ostende, LW (1989). The Talpidae (Insectivora, Mammalia) of Eggingen‐Mittelhart (Baden‐Württemberg, FRG) with special reference to the Paratalpa‐Desmanodon lineage. Stuttgarter Beiträge zur Naturkunde B, 152, 1.
11. Rzebik-Kowalska, B. (2014). Review of the Pliocene and Pleistocene Talpidae. Palaeontologia Electronica, 17(2).
12. Sánchez-Villagra MR, Menke PR (2005) The mole’s thumb: Evolution of the hand skeleton in talpids (Mammalia). Zoology. 108:3–12. https://doi.org/10.1016/j.zool.2004.07.006
13. Schwermann, A. H., He, K., Peters, B. J., Plogschties, T., & Sansalone, G. (2019). Systematics and macroevolution of extant and fossil scalopine moles (Mammalia, Talpidae). Palaeontology, 62(4), 661–676. https://doi.org/10.1111/pala.12422
